# Tregs biomimetic nanoparticle to reprogram inflammatory and redox microenvironment in infarct tissue to treat myocardial ischemia reperfusion injury in mice

**DOI:** 10.1186/s12951-022-01445-2

**Published:** 2022-06-03

**Authors:** Fangyuan Li, Daozhou Liu, Miao Liu, Qifeng Ji, Bangle Zhang, Qibing Mei, Ying Cheng, Siyuan Zhou

**Affiliations:** 1 Department of Pharmaceutics, School of Pharmacy, Air Force Medical University, Changle West Road 169, Xi’an, 710032 Shaanxi China; 2 Key Laboratory of Gastrointestinal Pharmacology of Chinese Materia Medica of the State Administration of Traditional Chinese Medicine, Department of Pharmacology, School of Pharmacy, Air Force Medical University, Xi’an, 710032 China

**Keywords:** Myocardial ischemia reperfusion injury, Regulatory T cells, Platelet membrane, Cyclosporin A, Reactive oxygen species, Macrophage

## Abstract

**Background:**

At present, patients with myocardial infarction remain an increased risk for myocardial ischemia/reperfusion injury (MI/RI). There lacks effectively method to treat MI/RI in clinic. For the treatment of MI/RI, it is still a bottleneck to effectively deliver drug to ischemic myocardium. In this paper, a regulatory T cells (Tregs) biomimetic nanoparticle (CsA@PPTK) was prepared by camouflaging nanoparticle with platelet membrane.

**Results:**

CsA@PPTK actively accumulated in ischemic myocardium of MI/RI mice. CsA@PPTK significantly scavenged reactive oxygen species (ROS) and increased the generation of Tregs and the ratio of M2 type macrophage to M1 type macrophage in ischemic myocardium. Moreover, CsA@PPTK significantly attenuated apoptosis of cardiomyocytes and reduced the infarct size and fibrosis area in ischemic myocardium. CsA@PPTK markedly decreased the protein expression of MMP-9 and increased the protein expression of CX43 in ischemic myocardium tissue. Subsequently, the remodeling of the left ventricle was significant alleviated, and heart function of MI/RI mice was markedly improved.

**Conclusion:**

CsA@PPTK showed significant therapeutic effect on MI/RI, and it has great potential application in the treatment of MI/RI.

**Graphical Abstract:**

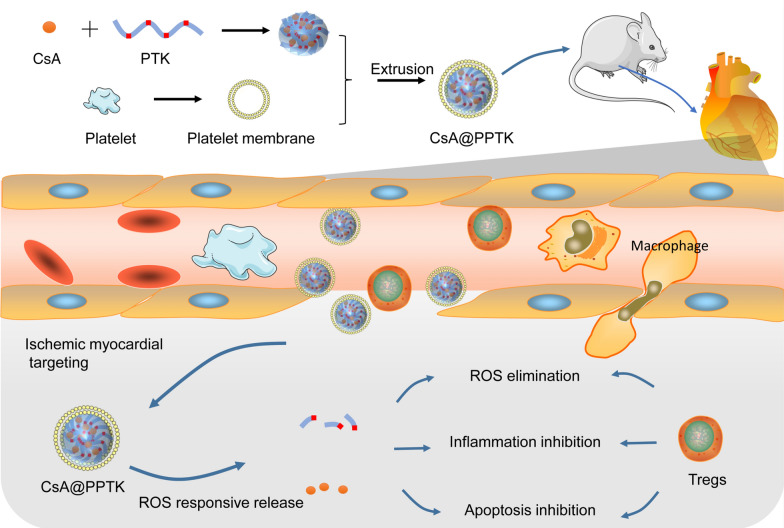

**Supplementary Information:**

The online version contains supplementary material available at 10.1186/s12951-022-01445-2.

## Background

Coronary heart disease such as blockage of coronary artery, acute myocardial infarction (AMI) and cardiac failure has become one of the leading causes of death in the world. More than 17.3 million people die from coronary heart disease every year [1]. Currently, the treatment of AMI is mainly focused on restoration coronary blood flow (reperfusion) through medications and/or revascularization procedures. Although reperfusion strategy is vital for survival [2], patients with AMI remain an increased risk for myocardial ischemia/reperfusion injury (MI/RI) [3–5]. There still lacks effectively method to treat MI/RI in clinic. Thus, exploring new strategies to treat MI/RI has important clinical significance and social benefits.

MI/RI is the result of multiple factors, including apoptosis of cardiomyocytes, reactive oxygen species (ROS) burst production, and inflammatory cell recruitment. During reperfusion, burst release of ROS and Ca^2+^ overload occur in cardiomyocytes, leading to the over-opening of mitochondrial permeability transition pore (mPTP). The over-opening of mPTP further induces more ROS release from mitochondria and the apoptosis of cardiomyocytes[6]. Finally, there forms a vicious cycle between the burst release of ROS and apoptosis of cardiomyocytes in ischemic myocardium tissue. Cyclosporine A (CsA) inhibits the over-opening of mPTP through binding with cyclophilin D in the mitochondrial inner membrane[7]. However, the solubility of CsA in water is poor [8], and the natural distribution of CsA in vivo lacks myocardial targeting. For ischemic myocardium targeting drug delivery system, long time retention in the blood and precise accumulation in ischemic myocardium are two bottlenecks that need to overcome.

There are some biomacromolecules such as GPIb-IX-V, αIIbβ3, intercellular cell adhesion molecule-1, p-selectin and α2β1 integrin on the surface of platelet. These biomacromolecules can bind with their corresponding receptors or collagens, including von willebrand factor, αVβ3 integrin and p-selectin glycoprotein ligand (PSGL-1), on vascular endothelial surface. Besides, von willebrand factor is highly expressed on the damaged vascular endothelial surface in ischemic myocardial tissue. Therefore, platelet binds damaged vascular endothelial with multiple binding sites. Actually, the cardiac blood flow velocity is fast, multiple binding sites enable platelets to quickly bind with damaged vascular endothelial in ischemic myocardium [9, 10]. This subsequently results in the recruitment of platelet in AMI area in vivo [1]. Thus, platelet and its membrane can be used as ischemic myocardium targeting material to guide nanoparticle to selective accumulation in ischemic myocardium [1, 11, 12]. When platelet membrane is coated on the surface of nanoparticle, the platelet membrane modified nanoparticle can not only actively accumulate in ischemic myocardium but also effectively reduce the non-specific phagocytosis of MPS and prolong the circulation time of nanoparticle in vivo [13, 14]. Besides platelet, regulatory T cell (Tregs) also actively target to ischemic myocardium. Tregs in ischemic myocardium tissue play the role in anti-apoptosis, anti-inflammatory, anti-oxidant and reduction of the remodeling of the left ventricle [15–18]. Thus, Tregs has protective effect on MI/RI [19]. MI/RI can be treated by increasing Tregs in ischemic myocardium tissue [20]. However, the preparation of Tregs is complex, and it is difficult for Tregs to be applied in clinical practice.

**Scheme 1:**
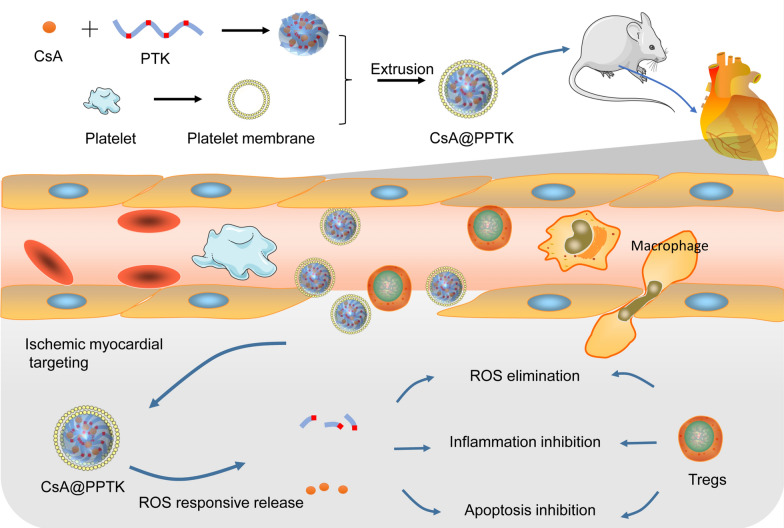
Schematic view of CsA@PPTK and its protective role on MI/RI mouse through ROS scavenging, anti-inflammatory and anti-apoptotic effect that mimic the Tregs

Inspired by the protective effect of Tregs on MI/RI and natural role of platelets in adhesion with damaged vascular endothelial in heart during myocardial infarct, a Tregs biomimetic nanoparticle was prepared by camouflaging nanoparticle with platelet membrane. Poly (5,5-dimethyl-4,6-dithio-propylene glycol azelate) (PTK) contains a large amount of thioketal which is sensitive to various types of ROS such as O_2_, ClO^−^ and H_2_O_2_ [21]. Thus PTK is able to scavenge these ROS. In this paper, CsA was encapsulated by PTK to form CsA@PTK. By using CsA@PTK as core and platelet membrane as shell, a Tregs biomimetic nanoparticle (CsA@PPTK) was prepared. CsA@PPTK mimics Tregs to perform a range of functions such as ischemic myocardium targeting, anti-inflammatory, anti-apoptotic and scavenging of reactive oxygen species. Finally, the remodeling of the left ventricle was significant alleviated, and heart function of MI/RI mice was markedly improved by CsA@PPTK. (Scheme 1).

## Materials and methods

### Materials

Cyclosporine A (CsA) was purchased from Shanghai Yuanye Biotechnology Co., Ltd (Shanghai, China). ROS test kit, mitochondrial membrane potential test kit (JC-1 solution) and RIPA lysate were purchased from Nanjing Beyotime Biotechnology Co. (Nanjing, China). Mitochondrial membrane permeability transition pore detection kit and mitochondrial superoxide red fluorescent probe were purchased from Shanghai Yisheng Biotechnology Co. (Shanghai, China). Antibodies against MMP-9, CX43, α-SMA and secondary antibodies were purchased from Servicebio (Wuhan, China). Antibody against CD86 was purchased from Thermo invitrogen Inc. (MA,USA). Antibody against CD206 was purchased from Cell Signaling Technology Inc. (Boston, USA). FITC anti-mouse CD4, APC anti-mouse CD25 and PE anti-mouse FoxP3 used for flow cytometry were purchased from Biolegend (CA, USA). DAPI was purchased from Thermo Fisher Scientific Inc. (MA, USA). TUNEL BrightRed Apoptosis Detection Kit, TNF-α and IL-1β ELISA kit were purchased from Servicebio (Wuhan, China). DiR and Cy7.5 dye were purchased from Ruitaibio (Beijing, China). H9c2 cardiomyoblast cell line was bought from Shanghai Institute of Cell Biology (Shanghai, China). All chemical reagents were obtained from reagent suppliers. Kunming mice and SD rats were provided by the Experimental Animal Center of Air Force Medical University (Xi’an, China). Animal experiments were approved by the Air Force Medical University Institutional Animal Care and Utilization Committee (No: IACUC-20190505).

### Synthesis of 5,5-dimethyl-4,6-dithio-azelaic acid

Acetone (6.1 mL) and 3-mercaptopropionic acid (3.5 mL) were mixed, and 3 drops of trifluoroacetic acid were added into above mixture. The reaction mixture was stirred overnight at room temperature. Next, the reaction mixture was cooled in ice bath for 4 h, the crystals (5,5-dimethyl-4,6-dithio-azelaic acid, TK) were collected. The product was dried in a vacuum drying oven for 24 h at 36 ℃. The structure of TK was identified by mass spectrometry (MS) and ^1^H NMR.

### Synthesis of poly(5,5-dimethyl-4,6-dithio-propylene glycol azelate)

Synthesis route of poly(5,5-dimethyl-4,6-dithio-propylene glycol azelate) (PTK) is showing in Additional file 1: Fig. S1. 1,3-Propanediol (670 μL) and Sc trifluoromethyl sulfonate (40 mg) were dissolved in 1 mL acetonitrile. The mixture was stirred at 40 ℃ for 30 min. Then, TK (2 g) was added into above mixture and stirred at 40 ℃ for 1 h. Next, the reaction temperature was raised to 50 ℃ under the protection of nitrogen. 1 h later, the temperature was raised to 90 ℃, and the reaction mixture was stirred for 24 h under the condition of vacuum. After the temperature of reaction mixture decreased to 25 ℃, chloroform (1 mL) and methanol (8 mL) were added into reaction mixture to precipitate the aimed product (PTK). After being separated, the precipitation was dried in a vacuum drying oven at 37 ℃ for 48 h. The structure of PTK was identified by ^1^H NMR and gel permeation chromatography (GPC).

### Preparation and characterization of CsA@PPTK

CsA@PTK nanoparticles were prepared by emulsion evaporation method. Briefly, PTK (10 mg) and CsA (2 mg) were dispersed into 20 mL polyvinyl alcohol (PVA, 0.5%) solution containing 2 mL dichloromethane. After mixture was performed ultrasound at 270 W for 3 min, the dichloromethane was removed by stirring the mixture for 12 h at room temperature. The nanoparticle suspension was filtered by using 0.4 μm membrane to remove free CsA. Ultrafiltration centrifuge tube (Millipore, USA) was used to remove PVA. Finally, CsA@PTK powder was obtained by lyophilization. Blank nanoparticle without CsA (@PTK) was prepared by the same method. Coumarin 6 labeled CsA@PTK (Coumarin 6@PTK) and Cy7.5 labeled CsA@PTK (Cy7.5@PTK) was prepared by using the same method as preparation for CsA@PTK except for adding Coumarin 6 (final concentration was 1 mg/mL) or Cy7.5 (final concentration was 0.5 mg/mL) into dichloromethane solution.

The platelet was isolated from rat plasma by differential centrifugation. With the help of ethylenediaminetetraacetic (EDTA) and prostaglandin E1, the platelet membrane was derived by using a repeated freeze–thaw process [22]. CsA@PPTK, @PPTK, Coumarin 6@PPTK and Cy7.5@PPTK were prepared by extrusion CsA@PTK, @PTK, Coumarin 6@PTK and Cy7.5@PTK with platelet membrane, respectively [23]. Size and zeta potential of nanoparticle were measured by using a Malvern ZEN 3600 Zetasizer. Hitachi S-4800 transmission electron microscope (TEM) was used to visualize the shape of nanoparticle. The protein on CsA@PPTK surface was studied by SDS-PAGE. The hemolytic effect of CsA@PPTK was investigated by using rat blood red cell.

### In vitro CsA release

Dialysis bags (MWCO: 3500 Da, Millipore) containing 5 mL suspension solution of different formulations were respectively immersed into 500 mL of PBS (pH 7.4) or PBS containing NaClO (an important ROS generated in ischemic myocardium under oxidative stress) under horizontal shaking at 37 °C. 50 μL solution in dialysis bag was taken out at different time point. CsA in release medium was analyzed by high performance liquid chromatography (HPLC, 2695/2996, Waters Corporation). The Dikma C_18_ column (4.6 mm × 250 mm, 5 μm) was used to separate CsA. Mobile phase is consisted with acetonitrile and water (v:v = 90:10). The column temperature was 55 ℃. The flow rate was 1 mL/min. The detection wavelength was 210 nm. The equation of the standard curve is *y* = 560.39*x*-288.15, R^2^ = 0.9999.

### Hypoxia reoxygenation injured H9c2 cells model

To prepare hypoxic culture medium, sodium lactate (1.12 g), 4-hydroxyethylpiperazine ethane sulfonic acid (0.475 g), sodium chloride (4.007 g), 2-deoxy-D-glucose (0.82 g), potassium chloride (0.59 g), sodium sulfate (0.093 g), hydrated calcium chloride (0.065 g) and magnesium chloride (0.05 g) were dissolved into 500 mL deionized water.

To set up hypoxia reoxygenation (H/R) injured H9c2 cells model, H9c2 cells were incubated in hypoxic culture medium under the condition of a hypoxic environment (95% N_2_ and 5% CO_2_) for 3 h at 37 °C. Next, the culture medium was replaced with DMEM solution containing CsA, CsA@PTK and CsA@PPTK, and H9c2 cells were cultured in a standard incubator with 5% CO_2_ in normal atmosphere for 4 h at 37 ℃. Then, the cell viability, intracellular ROS, mitochondrial ROS, the opening of mPTP and mitochondrial membrane potential of H9c2 cells were investigated.

### The effect of CsA@PPTK on the viability of H/R injured H9c2 cells

H/R injured H9c2 cells were incubated with MTT (20 μL/well, 5 mg/mL) for 4 h. The cell culture medium was replaced with dimethylsulfoxide (DMSO) solution. By using a microplate reader (Bio-Rad Laboratories, Richmond, CA, USA), the absorbance of DMSO solution was detected at 490 nm. The control cells were cultured in normoxic conditions with FBS-free DMEM solution for 7 h.

### The effect of CsA@PPTK on the intracellular ROS of H/R injured H9c2 cells

The intracellular ROS was detected by using 2′,7′-dichlorodihydrofluorescin diacetate (DCFH-DA). DCFH-DA probe was diluted with serum-free medium at the ratio of 1:1000. Then, H/R injured H9c2 cells were incubated with DCFH-DA solution for 20 min at 37 ℃. After DCFH-DA solution was removed, and the cells were washed with serum-free culture medium for 3 times. DAPI was used for nucleus staining. The cells were observed under fluorescent microscope (Olympus, Japan). The fluorescence intensity was semi-quantified with Image Pro software. Fluorescence intensity indicated ROS level in the cell. The stronger the fluorescence intensity is, the higher the ROS level is.

### The effect of CsA@PPTK on the mitochondrial ROS of H/R injured H9c2 cells

MitoSOX Indicator was diluted to 5 μM with HBSS. Then, the H/R injured H9c2 cells were incubated with diluted MitoSOX Indicator solution for 10 min at 37 ℃. DAPI was used for nucleus staining. The cells were observed by using laser scanning confocal microscopy (LSCM, Olympus, Japan). The fluorescence intensity was semi-quantified with Image Pro software. Fluorescence intensity indicated ROS level in mitochondria. The stronger the fluorescence intensity is, the higher the ROS level is.

### The effect of CsA@PPTK on the opening of mPTP of H/R injured H9c2 cells

H/R injured H9c2 cells were incubated with cell culture medium containing Calcein AM solution (1 mM) for 15 min, the cell culture medium was discarded. The fresh cell culture medium containing CoCl_2_ (80 mM) was added, and cells were incubated for 15 min at 37 °C. Next, HBSS/Ca (3.5 mL) was added into cell mixture. The cells were collected and re-suspended in buffer solution (400 μL) to carry out flow cytometric analysis. The stronger the fluorescence intensity is, the less the opening of mPTP is.

### The effect of CsA@PPTK on the mitochondrial membrane potential of H/R injured H9c2 cells

H/R injured H9c2 cells were incubated with fresh cell culture medium containing JC-1 (5 μg/mL) for 15 min at 37 °C. Then, the H9c2 cells were slightly rinsed 2 times with assay buffer. The fluorescent intensity of H9c2 cell solution were respectively detected at 530/590 nm (excitation/emission wavelength, red fluorescence) and 485/530 nm (green fluorescence) by fluorescence spectrophotometer (Hitachi, F-2700, Japan). The ratio between red fluorescent intensity and green fluorescent intensity was calculated. The greater the ratio value is, the higher the mitochondrial membrane potential is.

### Cellular uptake of CsA@PPTK in H/R injured H9c2 cells

H9c2 cells were seeded into 6 well plates (1 × 10^5^ cells/well). After hypoxia for 3 h, H9c2 cells were incubated with DMEM solution containing CsA@PTK or CsA@PPTK (30 μg CsA/mL) in a standard incubator with 5% CO_2_ in normal atmosphere for 0.5 h and 2 h. H9c2 cells were washed with PBS for 3 times. Then the cells were lysed by 100 μL RIPA lysis buffer. CsA in cell lysis was determined by HPLC. The protein content in cell lysis was determined by coomassie brilliant blue. The content of CsA in cell lysis was normalized by protein content in cell lysis.

To investigate the uptake pathway, H9c2 cells were seeded into 6 well plates (1 × 10^5^ cells/well). After hypoxia for 2 h, 2-deoxy-D-glucosesucrose (ATP depletion agent, 1 mg/mL), sucrose (inhibitor of clathrin-mediated uptake, 150 mg/mL), methyl-β-cyclodextrin (inhibitor of caveolae-mediated uptake, 0.005 mg/mL), colchicine (inhibitor of macropinocytosis, 0.8 mg/mL) were respectively added into H9c2 cells, and H9c2 cells were incubated for 1 h at 37 °C in hypoxic culture medium. Then, the hypoxic culture medium was replaced with DMEM solution containing CsA@PPTK (30 μg CsA/mL) and the same concentration of corresponding uptake inhibitor. H9c2 cells were incubated in a standard incubator with 5% CO_2_ in normal atmosphere for 2 h. The cells were collected and lysed. Finally, CsA in cell lysis was determined by using the same method as described above.

### Ex-vivo targeting ability study

The endothelium of abdominal aorta in SD rat was mechanically injured by using a balloon catheter. Then, the rats were euthanized, and their aortas were collected. The intact vessels and damaged vessels were cultured with Cy7.5-labeled CsA@PTK (Cy7.5@PTK) or Cy7.5-labeled CsA@PPTK (Cy7.5@PPTK) for 10 min. The vessels were washed with PBS for 3 times. The aortas was frozen and sectioned. The frozen sections were stained with CD31 antibody and DAPI. Finally, the sections were observed by fluorescence microscope.

### MI/RI mice model and the treatment

Kunming mice were anesthetized with 2% isoflurane inhalation. About 2 mm long vertical incision was cut at 2-3 mm away from the left sternal border. The chest wall muscle was separated. A small hole was made at intercostal space with a small hemostatic forceps to open the pleural membrane and pericardium. With the slightly open of hemostatic forceps, the heart was smoothly exposed out of the hole. By using a 6/0 silk suture, the left anterior descending coronary artery (LDA) was ligated at a site 3 mm from its origin. Only when the anterior wall of left ventricle (LV) became pale, the ligation was regarded as success. Heart was put back into chest immediately after ligation. The air in chest was extruded out, and the chest was closed with 4/0 suture. 30 min later, the 6/0 suture was carefully pulled out to restore the blood flow of LDA. 5 min before blood flow was restored, mice were given normal saline, CsA (1.25 mg/kg and 2.5 mg/kg), @PPTK ( 50 mg/kg), CsA@PTK (the equivalent CsA dose was 2.5 mg/kg) or CsA@PPTK (the equivalent CsA dose was 1.25 mg/kg and 2.5 mg/kg) via tail vein injection. The sham group performed the same surgical procedure except LDA was not ligated, and it were treated with normal saline.

### Biodistribution of CsA@PPTK in MI/RI mice

Acute myocardial ischemic Kunming mice were intravenously injected a single dose of Cy7.5 labeled CsA@PPTK (Cy7.5@PPTK) and Cy7.5 labeled CsA@PTK (Cy7.5@PTK) 5 min before reperfusion. 5 min and 24 h after injection of Cy7.5@PPTK, various organs (heart, liver, lung, spleen, and kidney) were collected and imaged by using Caliper IVIS Lumina II (Siemens, Germany). The heart tissue was then cut into five or six transverse sections and imaged again.

### Cardiac function of MI/RI mice

The cardiac function of MI/RI mice was assessed by transthoracic echocardiogram (Vevo 2100, VisualSonics, Canada). Animals were anesthetized by using a mixture of isoflurane and oxygen before undergoing transthoracic echocardiogram procedure at the 4 weeks and 10 weeks after reperfusion. Two dimensional images of LV were collected. Then, LV functional parameters such as left ventricular shortening fraction (FS) and left ventricular ejection fraction (LVEF) were calculated by using Vevo 2100 software.

### Tregs quantification

The heart from MI/RI mice was digested into a single cell suspension. Then, the cell suspension was incubated with a CD4-FITC antibody and CD25-APC antibody for 30 min. After that, the cell suspension was stained with FoxP3-PE antibody for 45 min. Finally, the CD4^+^ /CD25^+^/FoxP3^+^ cells were counted by flow cytometer.

### Histological and immunohistochemical analysis

MI/RI mice were sacrificed by intraperitoneal injection of over-dose pentobarbital sodium. Hearts were harvested. The contents of inflammatory cytokines such as IL-1β and TGF-β in heart were determined by ELISA kit. After being fixed in 4% paraformaldehyde, heart tissue was dehydrated by using gradient concentration of alcohol and embedded into paraffin. Then, heart tissue was cut into 4 μm thick sections for histological and immunohistological staining. For infarct size and fibrosis evaluation, masson trichrome staining was performed and total sections were scanned to acquire the whole images of heart horizontal planes. The fibrotic area was identified by using ImageJ software. The fibrosis was calculated as fibrotic area/total area in images. M1 type macrophage and M2 type macrophage in section of heart tissues were tracked by CD86 antibody and CD206 antibody, respectively. Then, fluorescence of CD206 and CD86 in section of heart tissues was observed by using LSCM. The expression of MMP-9, CX43 and α-SMA in section of heart tissues was observed by immunofluorescence method. TUNEL staining was used to track the apoptotic cardiomyocyte.

### In vivo safety of CsA@PPTK

CsA@PPTK (the equivalent CsA dose was 2.5 mg/kg) and normal saline were injected into Kunming mice via tail vein. 28 days later, the activated partial thrombin time (APTT), prothrombin time (PT) and fibrinogen level (Fbg) of in mice serum were analyzed by coagulation analyzer (Rayto, China) according to the manufacturer’s protocols. In addition, aspartate aminotransferase (AST), alanine aminotransferase (ALT), creatinine (CREA) and blood urea nitrogen (BUN) in mice serum were also detected by automatic biochemical analyzer (Chemray 800, Shenzhen, China). The major organs such as heart, liver, spleen, lung and kidney were fixed and stained by H&E. In order to further clarify the safety of @PTK, we studied the acute toxicity of @PTK in accordance with FDA Technical Guidelines for Non-clinical Safety Evaluation of Pharmaceutical Excipients [24]. Kunming mice were divided into 3 groups, and there were 10 mice in each group. @PTK (the dose of @PTK was 2 g/kg, 6 g/kg and 12 g/kg, respectively) was injected into Kunming mice via tail vein. The mice were observed for 2 weeks. Then, the mice were sacrificed, and the mice serum was isolated. The AST, ALT, CREA and BUN in mice serum were detected by automatic biochemical analyzer. The major organs such as heart, liver, spleen, lung, kidney and brain were fixed and stained by H&E.

### Statistical analysis

All results are expressed as means ± SD. Comparison between two groups was performed with two-tailed Student’s t test. The difference was considered statistically significant at the value of p < 0.05, 0.01 and 0.001.

## Results

### Characterization of TK and PTK

The molecular weight of TK is showing in Additional file 1: Fig. S2A, which was consistent with the theoretic molecular weight of TK. In addition, ^1^H NMR spectroscopy of TK showed peaks at 1.59, 2.90 and 2.68 ppm, it was assigned to Ha, Hb and Hc of TK, respectively (Additional file 1: Fig. S2B). Compared with TK, PTK displayed two new peak appeared at 4.18 and 1.98 ppm in ^1^H NMR spectroscopy, which were assigned to Hd and He of propanediol (Additional file 1: Fig. S3). The molecular weight of PTK was 20,505 Da determined by GPC (Additional file 1: Fig. S4).

### Characterization of CsA@PPTK

**Fig. 1:**
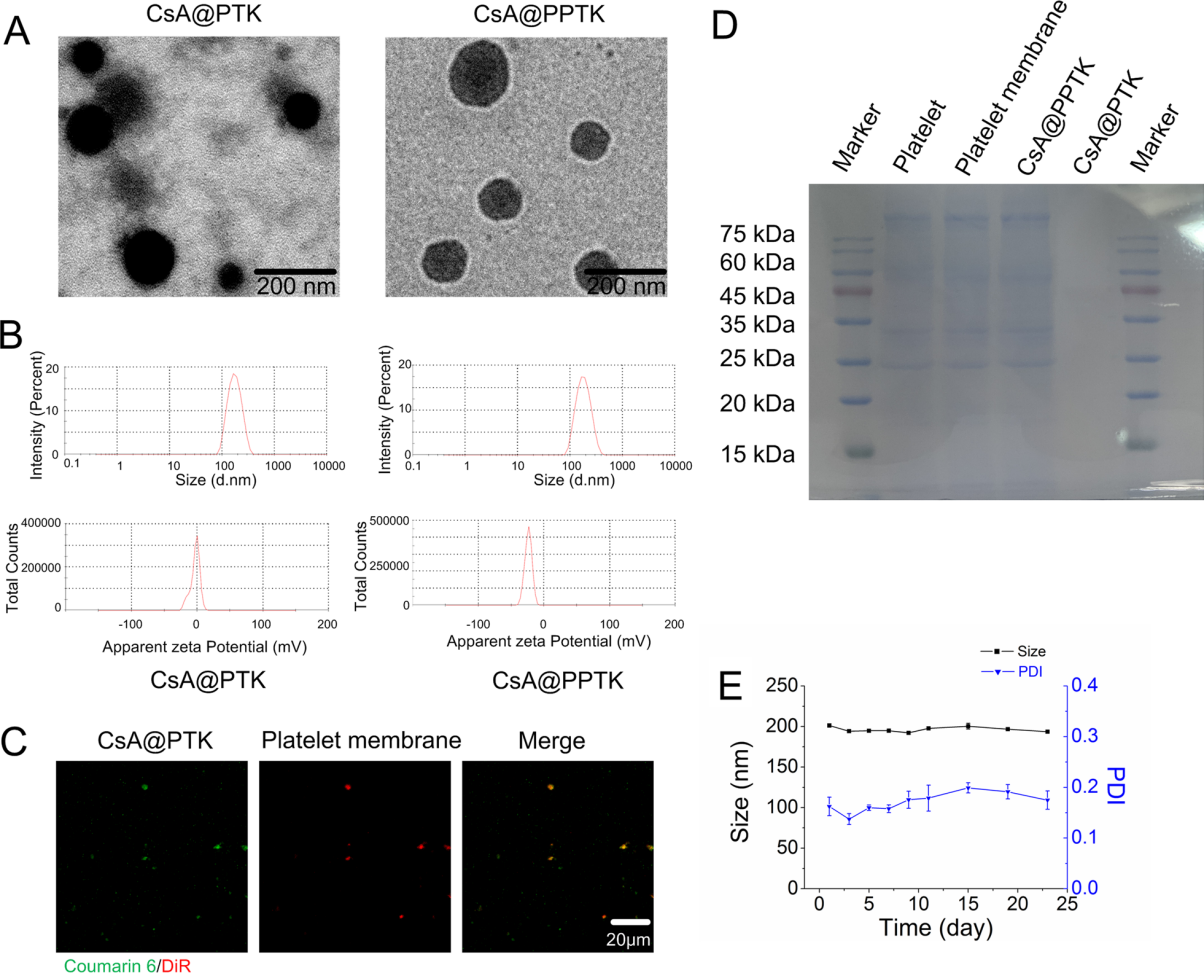
Characterizations of the CsA@PPTK. **A** TEM image. **B** Particle size distribution and zeta potential. **C** LSCM images of CsA@PPTK. (red: platelet membrane labeled with DiR, green: CsA@PTK labeled with Coumarin 6). **D** Protein band in platelet, platelet membrane, CsA@PPTK and CsA@PTK. **E** Stability of CsA@PPTK in PBS solution

The appearance of CsA@PTK and CsA@PPTK was spherical with good dispersion (Fig. 1A). Besides, CsA@PTK displayed a uniform structure from inside to outside, and CsA@PPTK showed obvious core–shell structure. This indicated that platelet membrane was coated on the surface of CsA@PTK. As compared with CsA@PTK, the size of CsA@PPTK did not show significant change. The zeta potential of CsA@PPTK was - 25 mV (Fig. 1B). The co-localization of the DiR-labeled platelet membrane (red) and coumarin 6 labeled CsA@PTK (green) further substantiated the successful coating of platelet membrane on the surface of CsA@PTK (Fig. 1C). SDS-PAGE electrophoresis revealed that the majority protein of platelet membrane was retained in CsA@PPTK (Fig. 1D). The drug loading of CsA in CsA@PPTK was 4.97%. As shown in Fig. 1E, the size and PDI of CsA@PPTK maintained stable over 23 days, and the size and PDI of CsA@PTK maintained stable within 9 days (Additional file 1: Fig. S5), indicating platelet membrane coating increased the stability of CsA@PTK.

### ROS scavenging ability and in vitro drug release of CsA@PPTK

**Fig. 2:**
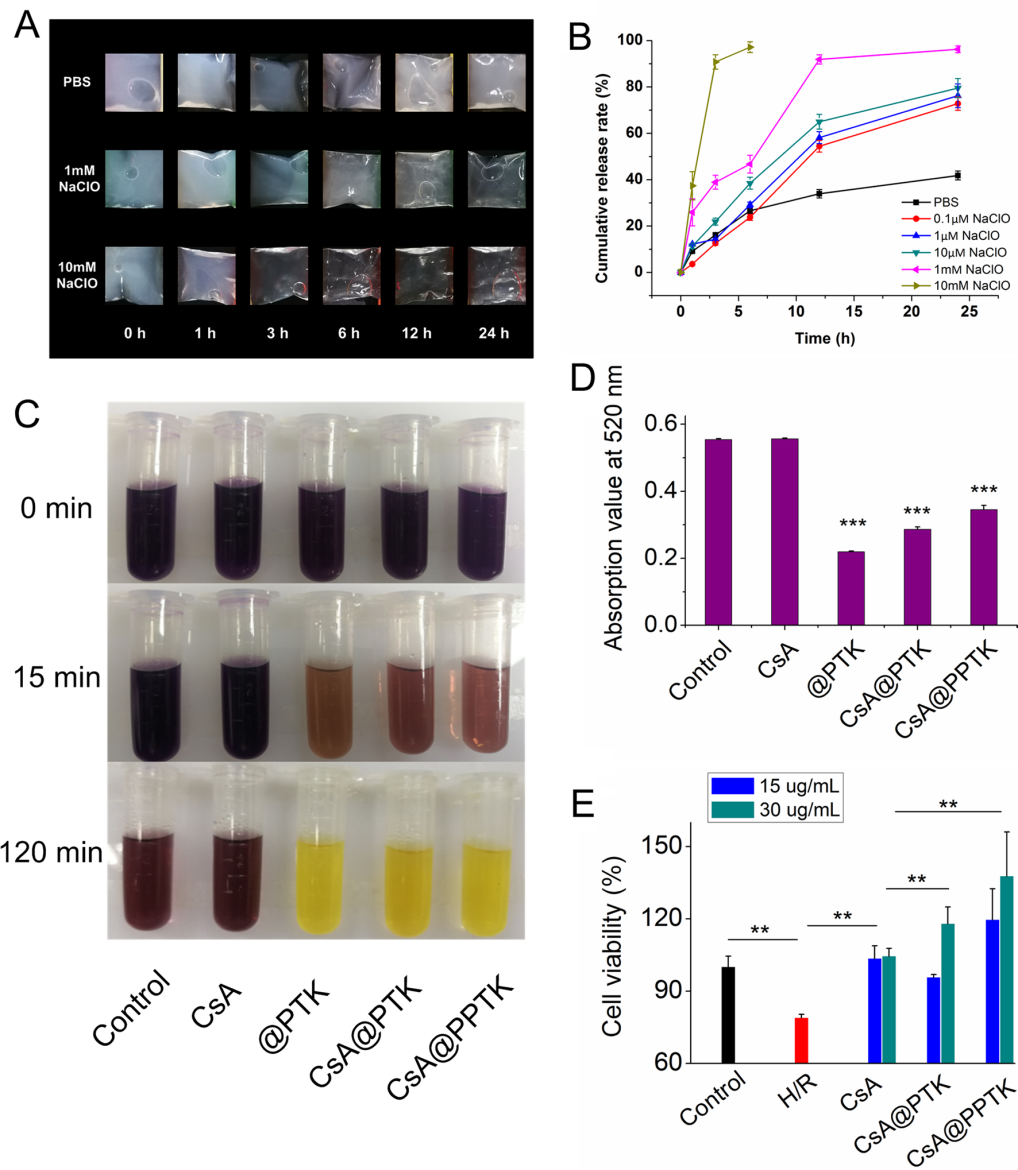
In vitro ROS responsibility of CsA@PPTK. **A** The appearance changes of CsA@PPTK in PBS (pH7.4) and PBS containing NaClO (pH7.4). **B** Cumulative drug release of CsA@PPTK in PBS (pH7.4) and PBS containing NaClO (pH7.4). n = 3, mean ± SD. **C** Color change of DPPH ethanol solution after treatment with CsA@PPTK. **D** Absorbance values of DPPH solution at 520 nm. n = 3, mean ± SD; ^***^p < 0.001 versus control group. **E** The effect of CsA@PPTK on viability of H/R injured H9c2 cells. n = 6, mean ± SD; ^**^p < 0.01

When CsA@PTK and CsA@PPTK was suspended in PBS, the suspension solution remained opalescence for 24 h. However, after being suspended in PBS solution containing 1 mM NaClO for 12 h, CsA@PTK and CsA@PPTK suspension solutions became clear. In PBS solution containing 10 mM NaClO, CsA@PTK and CsA@PPTK suspension solutions became clear within 3 h (Fig. 2A, Additional file 1: Fig. S6A). These results indicated that degradation rate of CsA@PTK and CsA@PPTK was dependent on NaClO concentration. The drug release results indicated that after incubation with PBS solution containing 0.1 μM NaClO at 37 ℃ for 24 h, about 70% CsA was released from CsA@PTK and CsA@PPTK. When concentration of NaClO in PBS solution increased to 1 mM, 95% CsA was released from CsA@PTK and CsA@PPTK within 24 h. Nevertheless, when CsA@PTK and CsA@PPTK was incubated with PBS, less than 40% CsA was released within 24 h (Fig. 2B, Additional file 1: Fig. S6B). These results demonstrated that CsA@PTK and CsA@PPTK released CsA in ROS-dependent manner, and platelet membrane coating did not affect the ROS-responsive characteristic of CsA@PPTK.

The in vitro ROS scavenging property of CsA@PPTK was assessed by DPPH assay [25]. When @PPTK, CsA@PTK and CsA@PPTK was incubated with DPPH solution for 15 min, the dark purple color of DPPH solution apparently faded as compared with DPPH or DPPH/CsA ethanol solution (Fig. 2C). Quantitative analysis showed that the absorbance value of DPPH solution containing @PPTK, CsA@PTK and CsA@PPTK significantly decreased with the prolongation of incubation time (Fig. 2D), revealing that @PPTK, CsA@PTK and CsA@PPTK scanvaged DPPH free radical.

### The protective effect of CsA@PPTK on H/R injured H9c2 cells

At first, the effects of @PPTK and CsA@PPTK on the activity of neonatal rat cardiomyocytes was investigated. The results indicated that @PPTK had no significant effect on the activity of neonatal rat cardiomyocytes within 48 h when @PPTK concentration ranged from 0.5 to 10 mg/mL (Additional file 1: Fig. S7A). CsA@PPTK showed no significant effect on the activity of neonatal rat cardiomyocytes within 48 h when CsA concentration ranged from 10 to 50 μg/mL (Additional file 1: Fig. S7B). In addition, 0.5 mg/mL, 1 mg/mL and 2 mg/mL @PTK improved the activity of H/R injured H9c2 cells (Additional file 1: Fig. S7C). It was reported that the optimized concentration of CsA to protect H/R injured H9c2 cells was 15 and 30 μg/mL [26]. The drug loading of CsA@PPTK was 4.97%. Therefore, 1 mg/mL @PTK was much higher than the maximum dose of @PTK which needed in the experiment, indicating that under the therapeutic doses, CsA@PTK exhibited no cytotoxicity on cardiomyocytes.

As shown in Fig. 2E, CsA, CsA@PTK and CsA@PPTK improved the activity of H/R injured H9c2 cells. The protective effect of CsA@PTK and CsA@PPTK on H/R injured H9c2 cells was similar to CsA when concentration of CsA was 15 μg/mL. However, when the concentration of CsA was 30 μg/mL, the protective effect of CsA@PTK and CsA@PPTK on H/R injured H9c2 cells was significantly stronger than that of CsA. This is because CsA@PPTK and CsA@PTK could scavenge ROS in H9c2 cells induced by H/R.

### The protective effect of CsA@PPTK on mitochondria of H/R injured H9c2 cells

Firstly, DCFH-DA staining demonstrated that there were a large amount of ROS in H/R injured H9c2 cells, indicating acute oxidative stress occurred after H/R (Additional file 1: Fig. S8A). However, ROS-positive cells and ROS level were significantly reduced in H/R injured H9c2 cells by using @PTK, CsA@PTK and CsA@PPTK (Additional file 1: Fig. S8B).

**Fig. 3:**
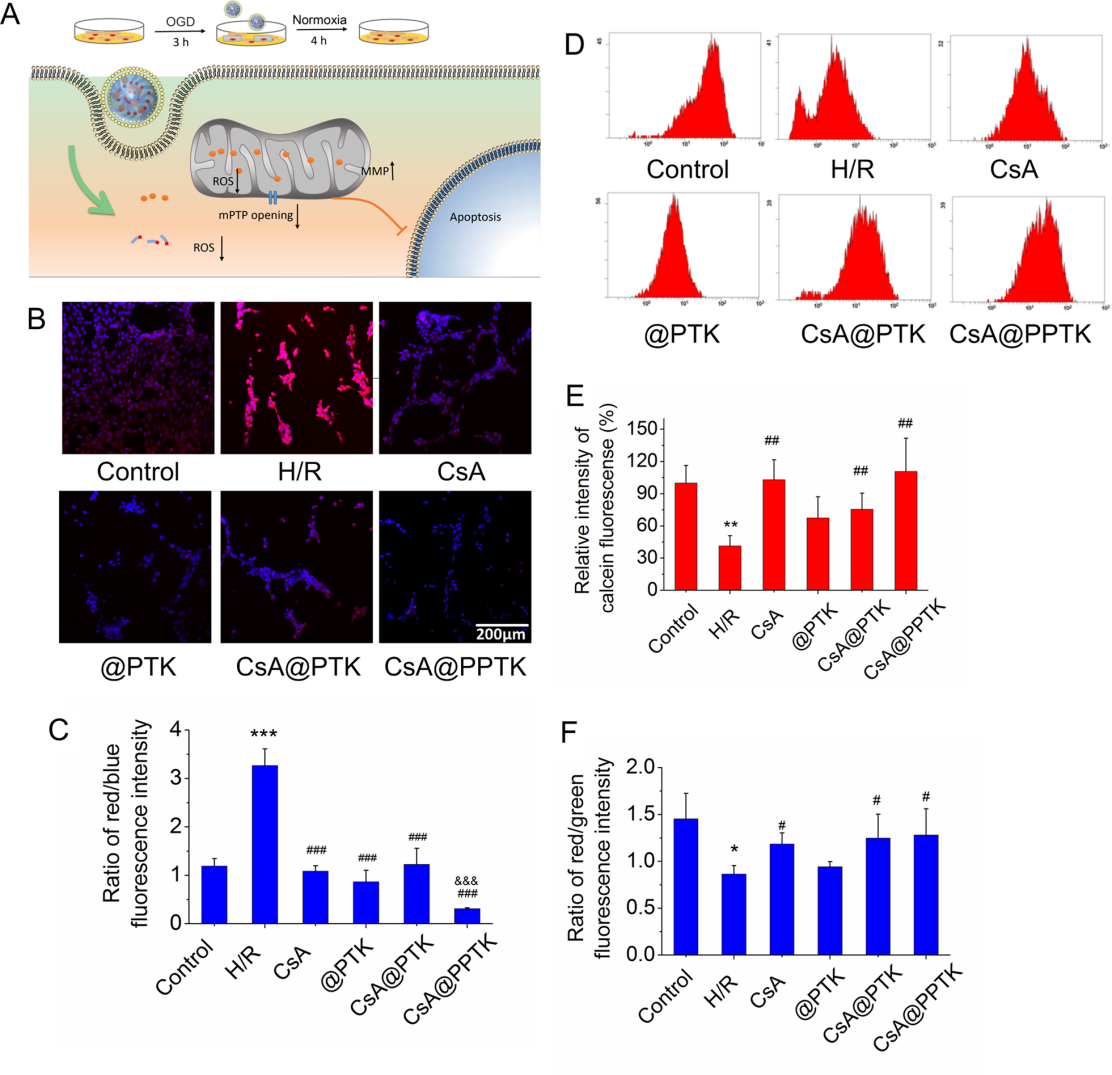
The protective effects of CsA@PPTK on H/R injured H9c2 cells. **A** Schematic diagram of the establishment of H/R injured H9c2 cell model. **B** Representative CLSM image of MitoSOX fluorescence in H/R injury H9c2 cells (red indicates MitoSOX, blue indicates cell nucleus). **C** Statistical results of MitoSOX fluorescence intensity. **D** Calcien AM fluorescence intensity measured in H/R injury H9c2 cells by flow cytometer. **E** Statistical results of Calcien AM fluorescence intensity. **F** Statistical results of the ratio of red to green fluorescence intensity in H/R injury H9c2 cells after JC-1 staining. n = 3, mean ± SD. ^*^p < 0.05, ^**^p < 0.01, ^***^p < 0.001 versus Control group. ^#^p < 0.05, ^##^p < 0.01, ^###^p < 0.001 versus H/R group. ^&&&^p < 0.001 versus CsA@PTK group

Secondly, the effect of CsA@PPTK on mitochondrial ROS is showing in Fig. 3B, C. As compared with control group, red fluorescence intensity was much stronger in H/R injured H9c2 cells, and red fluorescence intensity was much weaker in CsA, @PPTK, CsA@PTK and CsA@PPTK treated H/R injured H9c2 cells. The ratio of red fluorescence intensity to blue fluorescence intensity in H/R model group was significantly higher than that in control group, indicating that mitochondria produced a large amount of ROS after H9c2 cells was injured by H/R. As compared with H/R model group, the ratio of red fluorescence intensity to blue fluorescence intensity in CsA, @PPTK, CsA@PTK and CsA@PPTK treatment group was significantly reduced. Meanwhile, CsA@PPTK treated H/R injured H9c2 cells showed the lowest ratio of red fluorescence intensity to blue fluorescence intensity, indicating CsA, @PPTK, CsA@PTK and CsA@PPTK reduced mitochondrial ROS level in H/R injured H9c2 cells, and CsA@PPTK exhibited the strongest capacity on reducing mitochondrial ROS level in H/R injured H9c2 cells.

Thirdly, the effect of CsA@PPTK on the over-opening of mPTP in H/R injured H9c2 cells is showing in Fig. 3D, E. As compared with the normal group, the calcein fluorescence intensity in mitochondria in H/R injured H9c2 cells was significantly decreased, indicating that H/R significantly increased the opening of mPTP in H9c2 cells. As compared with the H/R model group, the calcein fluorescence intensity in mitochondria was significantly increased in CsA, CsA@PTK and CsA@PPTK treated H/R injured H9c2 cells, indicating that CsA, CsA@PTK and CsA@PPTK played a protective role against H/R injury by reducing the over-opening of mPTP in H/R injured H9c2 cells.

Finally, JC-1 staining was used to detect the mitochondrial membrane potential, and the results are showing in Fig. 3F. As compared with normal cultured H9c2 cells, the red fluorescence was obviously decreased and green fluorescence was obviously increased in H/R injured H9c2 cells, suggesting mitochondrial membrane potential was damaged during H/R in H9c2 cells. However, as compared with H/R model group, the ratio of red fluorescence intensity to green fluorescence intensity was significantly increased in CsA, CsA@PTK and CsA@PPTK treatment group, indicating that CsA, CsA@PTK and CsA@PPTK restored the damaged mitochondrial membrane potential.

### Cellular uptake of CsA@PPTK by H/R injured H9c2 cells

The uptake of CsA@PPTK by H9c2 cells is showing in Additional file 1: Fig. S9. The uptake of CsA@PTK and CsA@PPTK by normal and H/R injured H9c2 cells exhibited time-dependent manner. The uptake of CsA@PPTK in H/R injured H9c2 cells was much higher than that in normal H9c2 cells at 2 h. As compared with CsA@PTK, a large amount of CsA@PPTK was taken up by H/R injured H9c2 cells.

As shown in Additional file 1: Fig. S10, sucrose, colchicine, 2-deoxy-D-glucose and methyl-β-cyclodextrin showed no significant effect on the uptake of CsA@PPTK by H/R injured H9c2 cells. This suggested that the receptor-mediated endocytosis pathway did not involve in the uptake of CsA@PPTK by H/R injured H9c2 cells. As compared with the control group, the uptake of CsA@PPTK in H/R injured H9c2 cells significantly reduced at 4 ℃. In theory, adsorptive endocytosis is temperature dependent. The platelet membrane coated on the surface of CsA@PPTK is very similar with membrane of H/R injured H9c2 cells, which resulted in the strong adsorptive effect between CsA@PPTK and H/R injured H9c2 cells. Therefore, CsA@PPTK was taken up by H/R injured H9c2 cells mainly through adsorption endocytosis [27].

### The ex-vivo targeted of CsA@PPTK

**Fig. 4:**
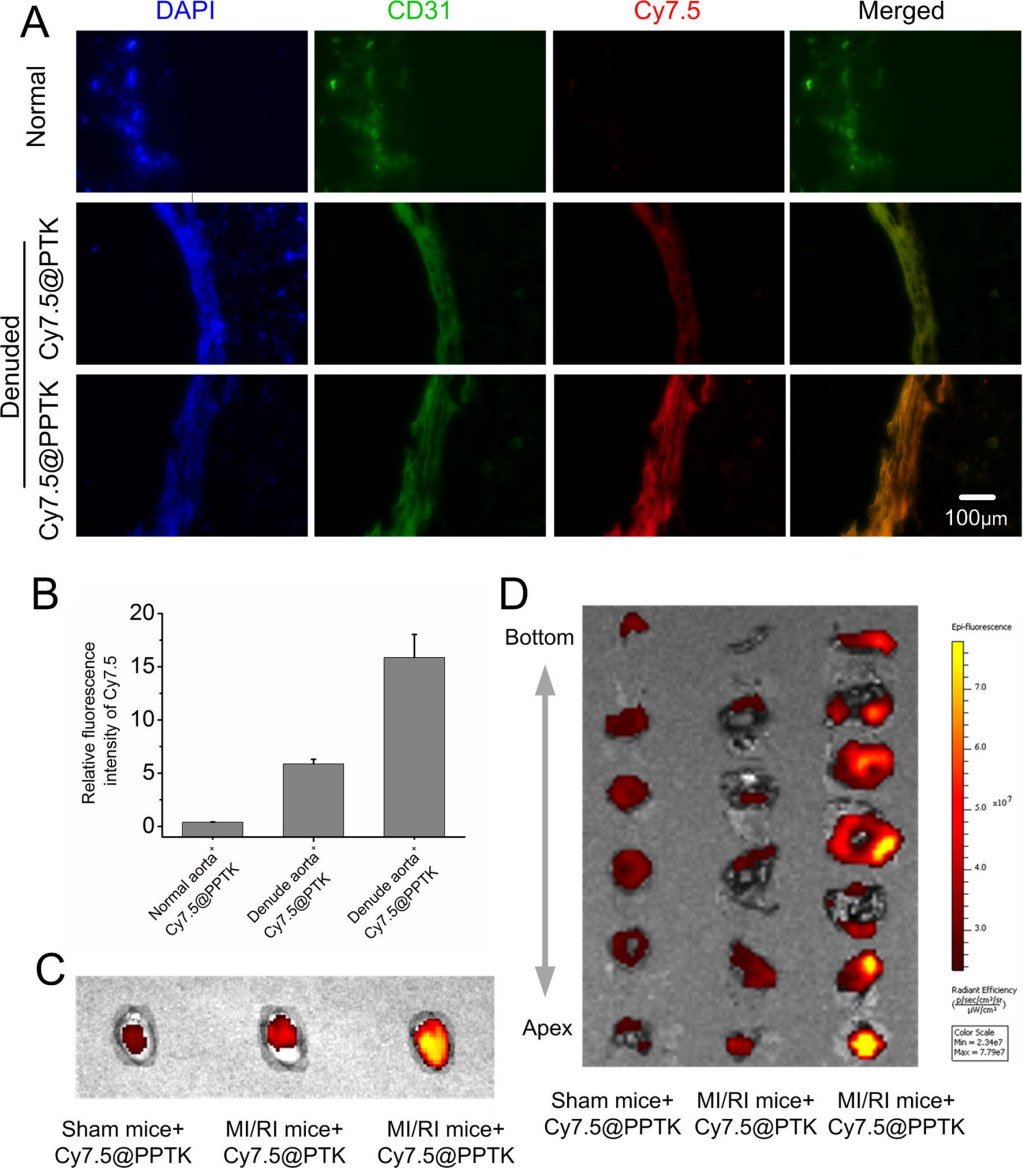
Targeted distribution of CsA@PPTK in ex vivo and in vivo. **A** Fluorescence of Cy7.5@PTK (red) or Cy7.5@PPTK (red) on normal and denuded aorta detected by fluorescent microscopic. Endothelium was marked with CD31, which presents green color. Cell nucleus was marked with DAPI, which presents blue color. **B** Statistical results of Cy7.5 fluorescence intensity in normal and denuded aorta. **C** Distribution of Cy7.5@PPTK in the hearts of sham and MI/RI mice at 24 h after administration. **D** The distribution of Cy7.5@PPTK in the heart slice of sham and MI/RI mice at 24 h after administration. n = 3, mean ± SD

The specific binding of Cy7.5@PPTK with endothelia injured aortic vessels is showing in Fig. 4A, B. The red fluorescence intensity of Cy7.5@PPTK in normal aortic vessels was very weak, indicating little amount of Cy7.5@PPTK was bound with normal aortic vessels. As compared with Cy7.5@PTK, the red fluorescence intensity of Cy7.5@PPTK in endothelia injured aortic vessels was much stronger, suggesting a large amount of Cy7.5@PPTK was bound with endothelia injured aortic vessels. The above data implied that CsA@PPTK could specifically bind with endothelia injured vessels in ischemic myocardium.

### In vivo targeting of CsA@PPTK

As shown in Additional file 1: Fig. S11, after acute myocardial ischemic mice were intravenously injected a single dose of Cy7.5@PPTK and Cy7.5@PTK at 5 min before reperfusion, a certain amount of Cy7.5@PPTK and Cy7.5@PTK was delivered to the myocardial tissue at 5 min after drug administration. The amount of Cy7.5@PPTK accumulated in MI/RI mice heart was significantly higher than that of Cy7.5@PTK. 24 h after drug administration, Cy7.5@PTK was mainly distributed in the liver and spleen, and little amount of Cy7.5@PTK was distributed in heart (Additional file 1: Fig. S12). However, As compared with Cy7.5@PTK, a large amount of Cy7.5@PPTK was distributed in the heart, while little amount of Cy7.5@PPTK was distributed in the liver of MI/RI mice. As shown in Fig. 4C, Cy7.5@PPTK was distributed in the whole heart when it was intravenously injected to sham mice. However, Cy7.5@PPTK was mainly distributed in downstream areas of the occluded coronary artery heart when it was intravenously injected to MI/RI mice. Cy7.5@PTK distributed in the whole heart of MI/RI mice. Next, the heart was cut into six or seven transverse sections from apex to atrium, and the distribution of Cy7.5@PPTK in transverse sections is showing in Fig. 4D. Cy7.5@PPTK distributed in all heart transverse sections of sham mice. Cy7.5@PTK also appeared in all heart transverse sections of MI/RI mice. However, Cy7.5@PPTK was mainly distributed in transverse sections of ischemic area especial in transverse section of apex in MI/RI mice heart. This indicated that Cy7.5@PPTK exhibited obvious ischemic myocardium targeting characteristic.

### In vivo therapeutic effect of CsA@PPTK on MI/RI mice

**Fig. 5:**
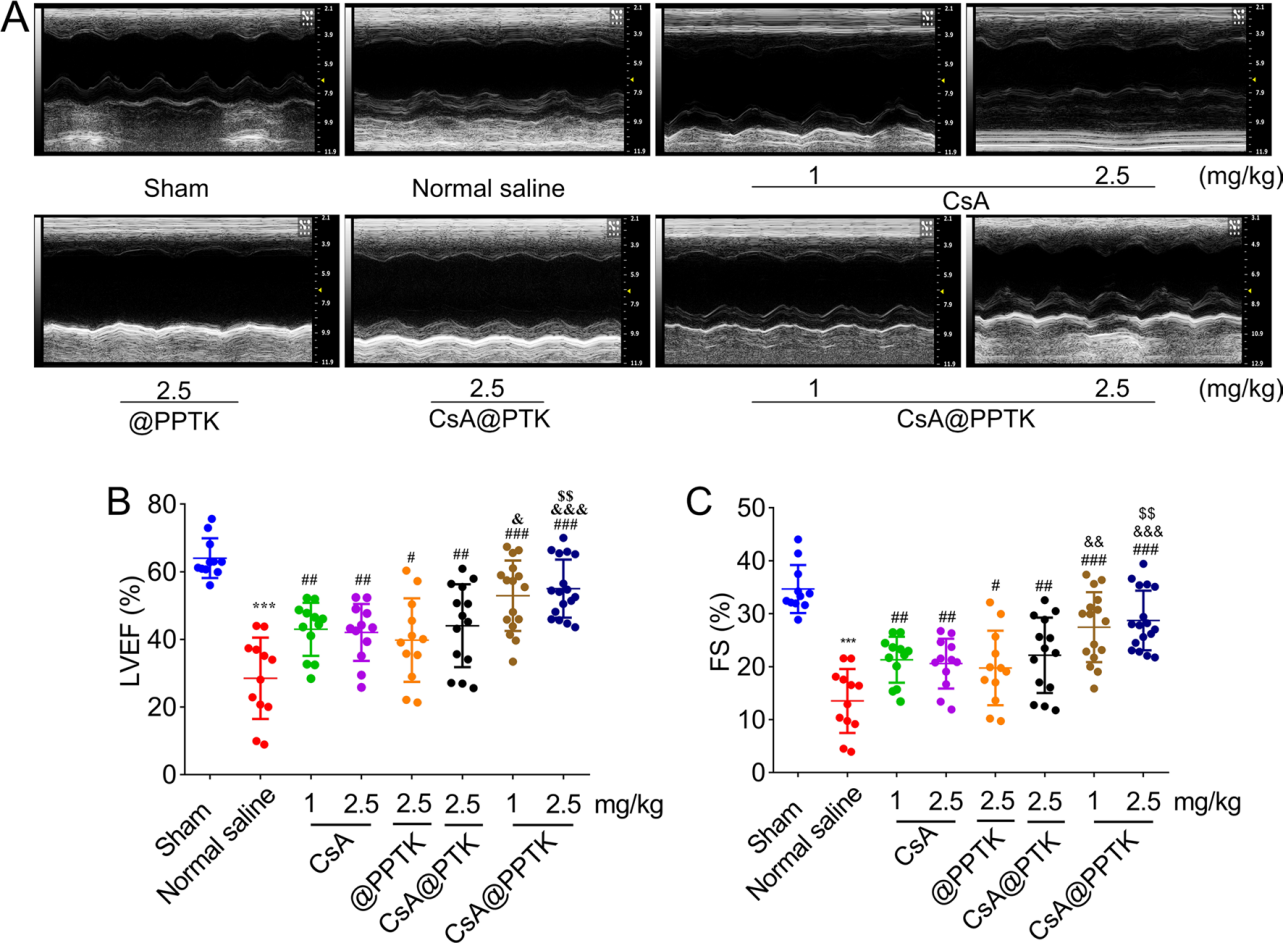
Left ventricular function of MI/RI mice at 28 days after administration of CsA@PPTK. **A** Representative echocardiography of MI/RI mice. **B** Statistical results of LVEF in different groups. **C** Statistical results of FS in different groups. n ≥ 12, mean ± SD; ^***^p < 0.001, compared with the sham group; ^#^p < 0.05, ^##^p < 0.01, ^###^p < 0.001, compared with normal saline group; ^&^p < 0.05, ^&&^p < 0.01, ^&&&^p < 0.001, compared with the same concentration of CsA group; ^$$^p < 0.01, compared with CsA@PTK group

On 28th day after reperfusion, echocardiography was applied to reveal the recovery of overall cardiac functions of MI/RI mice, and results are showing in Fig. 5. The mice in sham group showed regular and stable heart beats along with a large amplitude. The amplitude of heart beats was reduced and ventricular cavity volume was increased in normal saline treated MI/RI mice (Fig. 5A), suggesting an abnormal ventricular remodeling occurred. CsA and its preparations increased the amplitude of heart beats and decrease the ventricular volume of MI/RI mice as compared with normal saline. The left ventricular ejection fraction (LVEF) and fractional shortening (FS) reveal systolic function of the heart. Quantitative analysis revealed that LVEF and FS increased in CsA, @PPTK, CsA@PTK and CsA@PPTK treated MI/RI mice (Fig. 5B, C), suggesting the systolic function was improveed and abnormal ventricular remodeling was inhibited by using CsA, @PPTK, CsA@PTK and CsA@PPTK. As compared with normal saline, @PPTK obviously increased the heart function of MI/RI mice, indicating that ROS scavenging was an effective and necessary treatment method for MI/RI mice. CsA@PPTK treated mice showed the highest LVEF and FS in all group, signifying that ROS scavenging and anti-apoptosis improved heart function significantly. The echocardiography was also applied to reveal the recovery of overall cardiac functions on 70th day post MI/RI, and results showed that CsA@PPTK significantly improved the heart function at dose of 2.5 mg/kg (Additional file 1: Fig. S13).

The ROS level in ischemic myocardium 1 day after reperfusion is showing in Additional file 1: Fig. S14. The results indicated that as compared with sham group, the ROS level in ischemic myocardium in normal saline treated group (MI/RI model group) was significantly increased. CsA, @PPTK, CsA@PTK and CsA@PPTK markedly decreased ROS level in ischemic myocardium. CsA@PPTK treated group showed the lowest ROS level in ischemic myocardium.

**Fig. 6:**
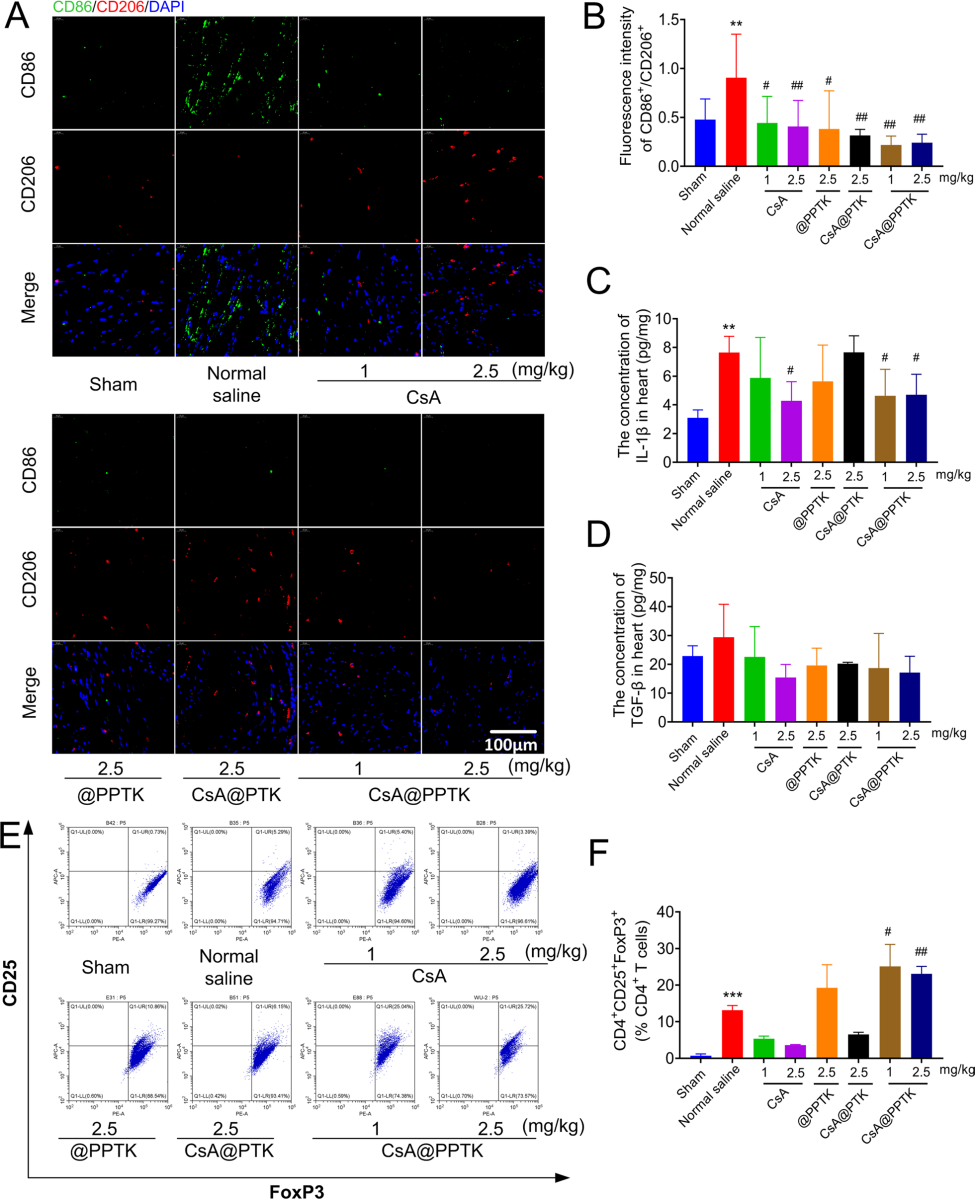
Effect of CsA@PPTK on immune microenvironment of myocardial tissue. **A** Representative images of CD86 staining (green) and CD206 staining (red) in myocardial tissue of MI/RI mice at 4 days after reperfusion. **B** The statistical results of fluorescence intensity of CD86/CD206. **C** IL-1β concentration in heart tissue of MI/RI mice at 4 days after reperfusion. **D** TGF-β concentration in heart tissue of MI/RI mice at 4 days after reperfusion. **E** Representative flow cytometer image of regulatory T cells (Tregs; CD4^+^CD25^+^FoxP3^+^) in heart tissue of MI/RI mice at 4 days after reperfusion. **F** Quantitative data of Tregs in heart tissue of MI/RI mice at 4 days after reperfusion. n = 3, mean ± SD; ^**^p < 0.01, ^***^p < 0.001 compared with sham group; ^#^p < 0.05, ^##^p < 0.01, compared with normal saline group

The phenotype of macrophage in infarcted heart tissue was detected on 4th day post reperfusion. As shown in Fig. 6A, B, as compared with normal saline treated group, the ratio of M2 type macrophages to M1 type macrophages obviously increased in heart tissue in CsA, CsA@PTK, @PPTK and CsA@PPTK treated groups. CsA@PPTK treated group exhibited the highest ratio of M2 type macrophages to M1 type macrophages in heart tissue. Besides, as compared with normal saline treated group, the number of M2 type macrophages and M1 type macrophages significantly reduced in heart tissue in CsA, CsA@PTK, @PPTK and CsA@PPTK treated groups. CsA@PPTK treated groups showed a minimum number of M2 type macrophages and M1 type macrophages in all groups. In addition, as compared with normal saline treated group, IL-1β and TGF-β level in heart tissue were decreased in CsA@PPTK treated MI/RI mice on 4th day after reperfusion (Fig. 6C, D). Flow cytometer results showed that Tregs in myocardial tissue significantly increased in the model group than that in sham group on 4th day after reperfusion, and Tregs in myocardial tissue was decreased after the treatment of CsA. CsA@PPTK significantly increased the number of Tregs in the infarct area as compared with model group (Fig. 6E, F).

**Fig. 7:**
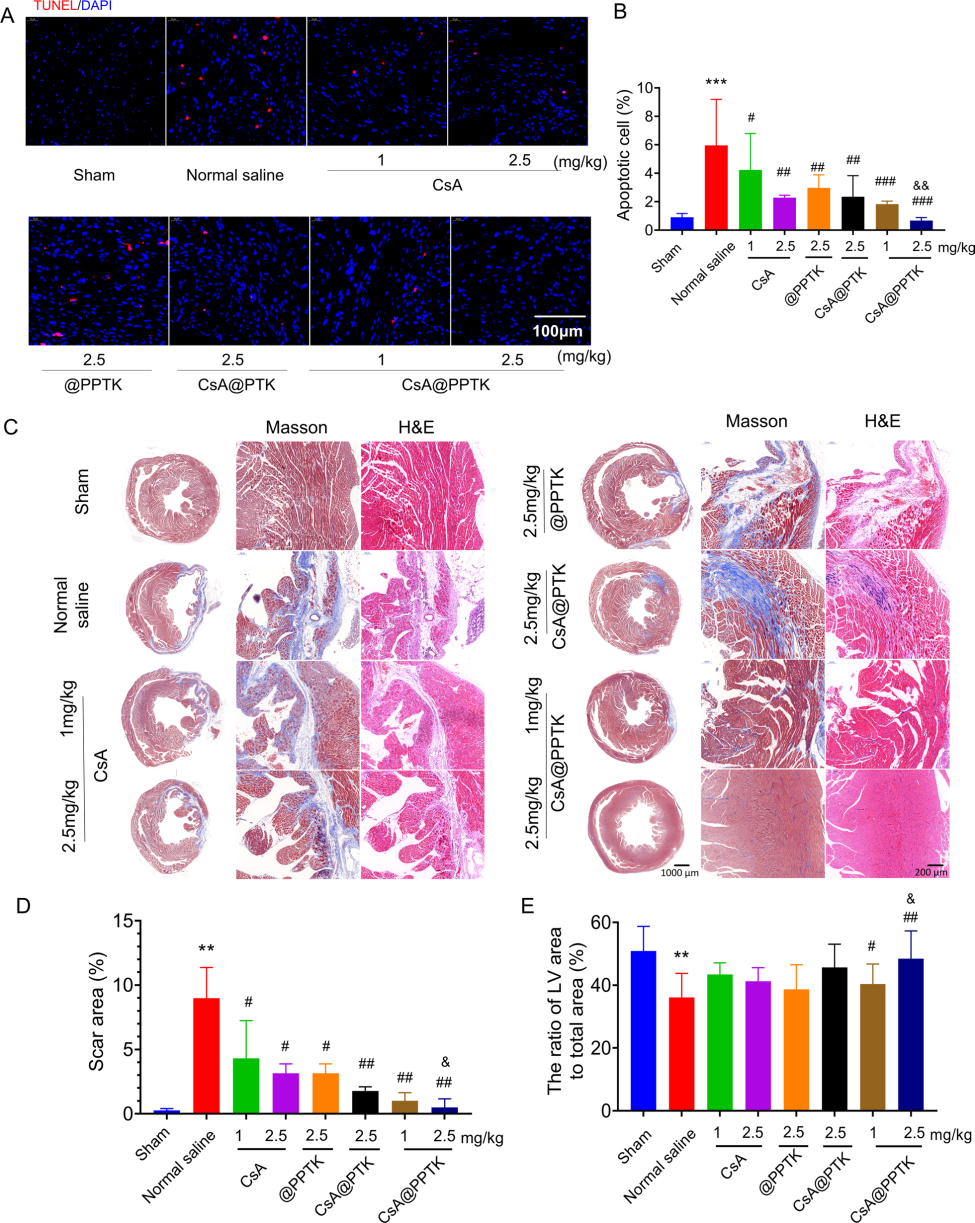
The protective effect of CsA@PPTK on MI/RI mice. **A** Representative images of TUNEL staining in myocardial tissue of MI/RI mice at 7 days after reperfusion. **B** The percentage of apoptotic cells. **C** Representative image of Masson and H&E staining of myocardial tissue of MI/RI mice at 28 days after reperfusion. **D** Statistical results of the scar area. **E** Statistical results of the LV area. n = 5, mean ± SD; ^**^p < 0.01, ^***^p < 0.001 compared with sham group; ^#^p < 0.05, ^##^p < 0.01, ^###^p < 0.001 compared with normal saline group; ^&^p < 0.05, ^&&^p < 0.01, compared with the same concentration of CsA group

TUNEL staining of infarcted heart tissue on 7th day after reperfusion is showing in Fig. 7A, B. As compared with normal saline treatment, CsA, @PPTK, CsA@PTK and CsA@PPTK significantly reduced the apoptosis of cardiomyocytes in ischemic area. CsA@PPTK treated group displayed a minimum number of apoptotic cardiomyocytes in ischemic area among all groups.

Masson trichrome staining and H&E staining of heart tissue are showing in Fig. 7C. In masson trichrome staining section, normal heart muscle is represented in red and fibrotic areas are represented in blue. As compared with sham group, the blue areas were increased in normal saline treatment group. Meanwhile, the ventricles were significantly dilated, and the walls of the ventricles were significantly thinner in the model group than that in sham group. The percentage of myocardial fibrosis area in myocardial section area is showing in Fig. 7D. The area of myocardial fibrosis in normal saline group was significant greater than that in sham group. CsA, @PPTK, CsA@PTK and CsA@PPTK significantly reduced myocardial fibrosis. As compared with the same dose of free CsA, CsA@PPTK significantly reduced myocardial fibrosis at the dose of 2.5 mg/kg. The percentage of left ventricular area to myocardial section area is showing in Fig. 7E. The percentage of left ventricular area to myocardial section area in normal saline group was marked smaller than that in sham group. CsA@PPTK significantly increased the percentage of left ventricular area to myocardial section area as compared with normal saline. As compared with the same dose of free CsA, CsA@PPTK significantly increased the percentage of left ventricular area to myocardial section area at the dose of 2.5 mg/kg. The above results demonstrated that CsA@PPTK strongly inhibited the cardiac remodeling and fibrosis, subsequently improved the cardiac function of MI/RI mice. These results were also consistent with the echocardiography data. In addition, a large number of inflammatory cells were infiltrated, and the structure of cardiomyocytes was unclear in normal saline group. The cardiomyocytes in sham group were orderly arranged and the cell structure was intact. In 2.5 mg/kg CsA@PPTK treatment group, the arrangement of cardiomyocytes was regular, and the inflammatory cell infiltration was decreased.

**Fig. 8:**
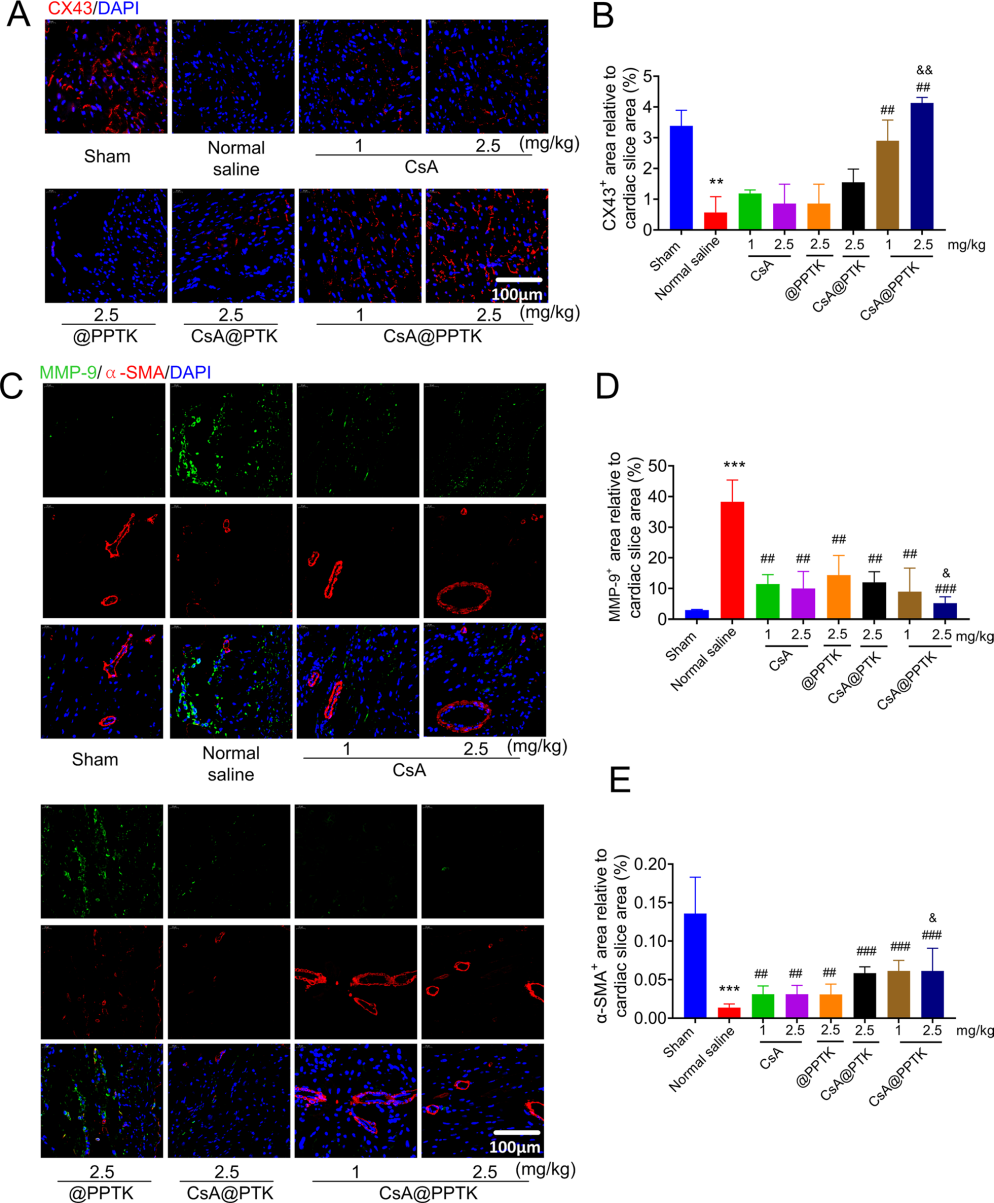
The effect of CsA@PPTK on expression of CX43, MMP-9 and α-SMA in myocardial tissue. **A** Representative images of CX43 staining in myocardial tissue of MI/RI mice at 28 days after reperfusion. **B** The statistical results of CX43 staining. **C** Representative images of MMP-9 staining and α-SMA staining in myocardial tissue of MI/RI mice at 28 days after reperfusion. **D** The statistical results of MMP-9 staining. **E** The statistical results of α-SMA staining. mean ± SD, ^**^p< 0.01, ^***^p < 0.001 compared with sham group; ^##^p < 0.01, ^###^p < 0.001, compared with normal saline group; ^&^p < 0.05, ^&&^p < 0.01, compared with the same concentration of CsA group

The effect of CsA@PPTK on the expression of CX43 in myocardium of MI/RI mice is showing in Fig. 8A, B. As compared with sham group, the expression of CX43 in the anterior wall of the left ventricle was significantly decreased in normal saline group. As compared with normal saline and same dose of CsA@PTK, CsA@PPTK significantly increased the expression of CX43 in the anterior wall of the left ventricle.

The effect of CsA@PPTK on the protein expression of MMP-9 in myocardium of MI/RI mice is showing in Fig. 8C, D. The protein expression of MMP-9 in the left ventricle anterior wall was significantly increased in normal saline group than that in sham group. CsA, @PPTK, CsA@PTK and CsA@PPTK significantly reduced the protein expression of MMP-9 in the left ventricle anterior wall of MI/RI mice as compared with normal saline. CsA@PPTK markedly reduced the expression of MMP-9 in the left ventricle anterior wall than the same dose of CsA@PTK. Interestingly, the arteriole density in the infarct area was also increased (Fig. 8E) in CsA@PPTK treatment group, indicating that blood vessels were preserved in infarct area by using CsA@PPTK.

### Preliminary safety assessment of CsA@PPTK

CsA@PPTK did not cause hemolysis of red blood cells in vitro (Additional file 1: Fig. S15). As shown in Additional file 1: Fig. S16, on 28 days after intravenous injection of CsA@PPTK and normal saline, the APTT, PT and Fbg in CsA@PPTK group showed no significant difference from that in normal saline group, indicating CsA@PPTK did not cause blood coagulation. Between CsA@PPTK group and normal saline group, the ALT and AST activities, BUN and CREA contents in mice serum also showed no significant difference (Additional file 1: Fig. S17). Meanwhile, no histopathological changes were observed in major organ tissue after injection of CsA@PPTK (Additional file 1: Fig. S18). In addition, it was found that there was no dead mice, and no histopathological changes were observed in major organ tissue after intravenous injection of @PTK to mice at the dose of 2 g/kg, 6 g/kg and 12 g/kg (Additional file 1: Fig. S19). The level of AST, ALT, BUN and CRE in mice serum was all within normal range after intravenous injection of @PTK to mice at the dose of 2 g/kg, 6 g/kg and 12 g/kg (Additional file 1: Fig. S20). The above results indicated that @PTK was none toxicity even at high dose, and @PTK was safe at the therapeutic dose according to the FDA Technical Guidelines for Non-clinical Safety Evaluation of Pharmaceutical Excipients[24].

## Discussion

MI/RI is actually the result of multiple factors including apoptosis of cardiomyocytes, oxidative stress and inflammatory reaction. The increase of ROS leads to apoptosis of cardiomyocytes, inflammatory cell recruitment and inflammatory reaction [28, 29]. Thus, ROS is an important factor in MI/RI. Scavenging ROS has been proved to have protective effect on MI/RI.

In recent years, ROS-responsive materials have been paid more attention in drug delivery system. This kind of material can not only be degraded but also release drug at sites with high ROS level. At present, ROS-responsive materials are mainly based on phenylboric acid and thioketal. Thioketal bond can be broken by various ROS such as potassium superoxide (KO_2_), H_2_O_2_, hydroxyl radical (∙OH), hypochlorite (ClO^−^) and peroxynitrite (ONOO^−^) [30]. In this study, based on thioketal, a ROS-responsive material PTK was synthesized. PTK contained a large amount of thioketal bonds. The drug release experiment indicated CsA@PPTK released 20% of the total loaded drug in PBS solution within 6 h, which is mainly caused by passive diffusion. Biodistribution experimental results indicated that a certain amount of CsA@PPTK accumulated in ischemic myocardial tissue 5 min after intravenous injection, indicating little amount of CsA would be prematurely released from CsA@PPTK in blood circulation. When CsA@PPTK reached the ischemic myocardium, PTK was broken by ROS in ischemic myocardium. Subsequently, CsA was rapidly released. This was beneficial for the treatment of reperfusion injury. In addition, CsA@PTK and CsA@PPTK released CsA in ROS-dependent manner. This suggested the high ROS level microenvironment induced by ischemia reperfusion would facilitate the release of CsA from CsA@PPTK. The cell experiment showed that the ROS level in H/R injured H9c2 cells was decreased by using @PPTK, CsA@PTK and CsA@PPTK. Besides, CsA, CsA@PTK and CsA@PPTK reduced the over-opening of mPTP and restored the mitochondrial membrane potential of H/R injured H9c2 cells. The above results demonstrated that CsA@PPTK protected H9c2 cells from H/R injury by strongly blocking the over-opening of mPTP and eliminating ROS in H/R damaged H9c2 cells. PTK could scanvage ROS in H/R injured H9c2 cells.

After being intravenously injected, the amount of CsA@PPTK accumulated in liver of MI/RI mice was significantly reduced as compared with CsA@PTK. This demonstrated that platelet membrane coated on the surface of nanoparticle markedly reduced the phagocytosis of nanoparticle by mononuclear phagocytic system (MPS) [14]. Besides, the transmembrane protein GPIV and GPIX on the platelet membrane could bind with the damaged blood vessels. Thus, the ex vivo experiment results indicated CsA@PPTK could specifically bind with endothelial damaged blood vessel wall. The integrin-related proteins CD9 and CD81 on the platelet membrane could increase the uptake of CsA@PPTK by cardiomyocytes [31, 32]. This indicated that CsA@PPTK could actively target to cardiomyocytes in ischemic myocardium of MI/RI mice. Therefore, after being intravenously injected to MI/RI mice, CsA@PPTK mainly distributed in apical tissue with the most severe cardiac ischemia. In addition, the in vitro experimental results showed that CsA@PPTK quickly released CsA at the present of ROS. The in vivo experimental results indicated there was a large amount of ROS in ischemic myocardium tissue after reperfusion. Therefore, CsA@PPTK could fast release CsA and reduced the over-opening of mPTP of cardiomyocytes in cardiac ischemia area. At the same time, CsA@PPTK strongly scavenged ROS in ischemic myocardium tissue. Finally, CsA@PPTK alleviated apoptosis of cardiomyocytes and decreased myocardial infarction size in MI/RI mice.

Phase one inflammatory reaction induced by M1 type macrophages is closely related to the generation of ROS, which is known as activation of inflammatory macrophages and facilitating recruitment of inflammatory cell to infarct area [33, 34]. The phenotype change of macrophages plays a key role in the progress of tissue repair. The increased ratio of M2 to M1 type macrophage is of benefits to the reduction of chronic inflammation and fibrosis in cardiac ischemia tissue. Some studies have also found that timely transformation of M1 type macrophages to M2 type macrophages can effectively increase the repair of infarcted myocardial tissue [35, 36]. In vivo experimental data indicated that CsA@PPTK obviously increased the ratio of M2 type macrophages to M1 type macrophage in heart tissue. At the same time, the number of M1 type macrophages and M2 type macrophages markedly decreased in CsA@PPTK treated group, indicating the damaged myocardium tissue was repaired by CsA@PPTK.

IL-1β is a pro-inflammatory factor. Our study showed that MI/RI significantly increased the level of IL-1β in heart, while CsA, @PPTK, CsA@PTK and CsA@PPTK reduced the level of IL-1β. CsA@PPTK displayed the most greatest effect on the reduction of IL-1β. These results indicated that CsA@PPTK displayed anti-inflammatory effect, which was consistent with the results of immunofluorescence staining. This was resulted from following three reasons. Firstly, CsA@PPTK remodeled the oxidized microenvironment of MI/RI by scavenging ROS in cardiac ischemia area. Secondly, CsA@PPTK reduced apoptosis of cardiomyocytes by inhibiting the over-opening of mPTP. The vicious cycle between the burst release of ROS and apoptosis of cardiomyocytes in ischemic myocardium tissue was blocked. Finally, CsA inhibited the immune response mediated by T lymphocytes, and reduced the release of various cytokines such as IL-2, IL-3 and IFN-γ. Consequently, recruitment of inflammatory cells to damaged myocardium tissue was weakened and inflammatory reaction was alleviated. The above data demonstrated that CsA@PPTK alleviated MI/RI and recovered the heart function through reducing systemic inflammatory reaction.

TGF-β overexpression induces the transformation of cardiac fibroblasts into myofibroblasts, leading to myocardial fibrosis and cardiac remodeling [37]. Our study showed that TGF-β level increased after MI/RI in mice. As compared with normal saline, CsA@PPTK reduced the protein express of TGF-β. The level of TGF-β in the CsA@PPTK group showed no significant difference from sham group. The above data suggested that CsA@PPTK could reduce cardiac remodeling by reducing protein express of TGF-β in myocardial infarction tissue.

Tregs inhibited the activity of cytotoxic T cells and M1 type macrophage, and then suppressed local inflammatory response and systemic cytotoxic response induced by myocardial infarction. Therefore, Tregs reduced ventricular remodeling and improved cardiac function [38, 39]. The experimental results showed that the number of Tregs in the myocardial infarction area increased significantly 4 days after reperfusion, which was due to the fact that circulating Tregs infiltrated into the myocardial tissue through vascular wall in the early stage of myocardial injury. CsA displayed an inhibitory effect on Tregs. Thus, a significant decrease in Tregs in cardiac tissue was observed after free CsA was administered. Studies have found that ROS scavenger can increase the number of Tregs. Therefore, CsA@PPTK increased Tregs in ischemic myocardium tissue by scavenging ROS in myocardial infarction tissue. In a word, by reprograming Tregs generation, the ratio of M2 type macrophage to M1 type macrophage and oxidative microenvironment, CsA@PPTK alleviated inflammatory reaction and subsequently reduced myocardial fibrosis and the remodeling of left ventricle.

MMP-9 is closely related to the remodeling of left ventricle. Elevated MMP-9 levels have been found in ventricular remodeling tissue after AMI [40, 41]. The activity of MMP-9 is positively correlated with the severity of AMI, and the mortality of MI/RI animals was significantly increased with the increase of MMP-9 expression. The experimental data demonstrated that CsA@PPTK markedly attenuated left ventricular remodeling by reducing MMP-9 protein expression in left ventricle anterior wall of MI/RI mice.

CX43 is the most important protein in the gap junction of the left ventricular muscle [42]. The cardiac electrochemical impulse is mainly transmitted to the cardiomyocytes of the left ventricle through CX43 to maintain the rhythmic contraction of the left ventricle. Under normal circumstances, CX43 mainly exists as a phosphorylated status. When myocardial ischemia occurs, the expression of CX43 is reduced, and CX43 protein is dephosphorylated, which eventually induces arrhythmias. The experimental results showed that CsA@PPTK significantly increased the expression of CX43 as compared with free CsA and CsA@PTK, indicating CsA@PPTK greatly restored the activity of cardiomyocytes and cardiac function.

Ikeda et al. prepared a CsA-loaded PLGA nanoparticles (CsA-NPS) to treat MI/RI in mice [43]. CsA could be delivered to ischemic myocardium by intravenous injection of CsA-NPS. CsA-NPS enhanced the protective effect of CsA on ischemia myocardium by inhibiting the over-opening of mPTP and reducing the left ventricular remodeling in mice. Zhang et al. used mitochondrial targeted peptide SS31 modified PLGA to deliver CsA to treat MI/RI in mice [26]. The results showed that as compared with PLGA nanoparticles, SS31 modified PLGA nanoparticles delivered more amount of CsA to the deep ischemic region of myocardium and attenuated the apoptosis of cardiomyocytes by inhibiting the over-opening of mPTP in cardiomyocytes. As compared with previous studies, this study has the following three innovations. (1) CsA@PPTK actively accumulated in ischemic myocardium tissue by platelet membrane encapsulation, improving the targeting of CsA to ischemic myocardium and reducing the accumulation of CsA@PPTK in liver. (2) CsA was encapsulated by the ROS responsive material PTK, and CsA was released from CsA@PPTK in the high ROS environment of ischemic myocardium. Subsequently, CsA played a protective effect on ischemic myocardium by inhibiting the over-opening of mPTP. (3) PTK significantly scavenged the ROS in myocardial ischemia tissue, producing a synergistic effect with CsA on the treatment of MI/RI.

Although CsA@PPTK showed significant therapeutic effect on MI/RI in mice, there were some limitations of the current work. (1) The pharmacokinetics and toxicity of CsA@PPTK should be thoroughly studied in animal. (2) The drug-drug interaction should be taken into consideration if CsA@PPTK could be used in clinic in the future. At present, patients with acute myocardial infarction generally take antiplatelet drugs for a long time, and some antiplatelet drugs such as glycoprotein (GP) IIb/IIIa receptor inhibitors may weaken the binding of CsA@PPTK with the injured endothelial cell in vivo.

## Conclusion

CsA@PPTK released CsA in ROS-dependent manner, and it actively accumulated in ischemia myocardium after intravenous injection. CsA@PPTK significantly alleviated the remodeling of the left ventricle and enhanced heart function through reprograming inflammatory and redox microenvironment and attenuating apoptosis of cardiomyocytes at infarct heart tissue. CsA@PPTK has great potential application in the treatment of MI/RI.

## Supplementary Information

**Additional file 1.** Additional figures.

## Data Availability

All data generated or analyzed during this study are included in this published article and its supplementary information file.

## Abbreviations

ALT: Alanine aminotransferase
AMI: Acute myocardial infarction
APTT: Activated partial thrombin time
AST: Aspartate aminotransferase
BUN: Blood urea nitrogen
LSCM: Laser scanning confocal microscopy
CREA: Creatinine
CsA: Cyclosporine A
DCFH-DA: 2′,7′-Dichlorodihydrofluorescin diacetate
Fbg: Fibrinogen
FS: Shortening fraction
H/R: Hypoxia reoxygenation
LDA: Left anterior descending coronary artery
LV: Left ventricle
LVEF: Left ventricular ejection fraction
MI/RI: Myocardial ischemia/reperfusion injury
PSGL-1: P-selectin glycoprotein ligand
PT: Prothrombin time
PTK: Poly (5,5-dimethyl-4,6-dithio-propylene glycol azelate)
PVA: Polyvinyl alcohol
ROS: Reactive oxygen species
TEM: Transmission electron microscope
Tregs: Regulatory T cells
