# Supplementary material for: Tregs biomimetic nanoparticle to reprogram inflammatory and redox microenvironment in infarct tissue to treat myocardial ischemia reperfusion injury in mice
Source: J Nanobiotechnology. 2022 Jun 3;20:251. doi: 10.1186/s12951-022-01445-2 (PMC9164893; doi:10.1186/s12951-022-01445-2)
Supplement: Supplementary file 1 — Additional file 1. Additional figures. [file 12951_2022_1445_MOESM1_ESM.docx]

Supporting Information

**Tregs Biomimetic Nanoparticle to Reprogram Inflammatory and Redox Microenvironment in Infarct Tissue to Treat Myocardial Ischemia Reperfusion Injury in Mice**

Fangyuan Li1, Daozhou Liu1, Miao Liu, Qifeng Ji, Bangle Zhang, Qibing Mei, Ying Cheng*, Siyuan Zhou*


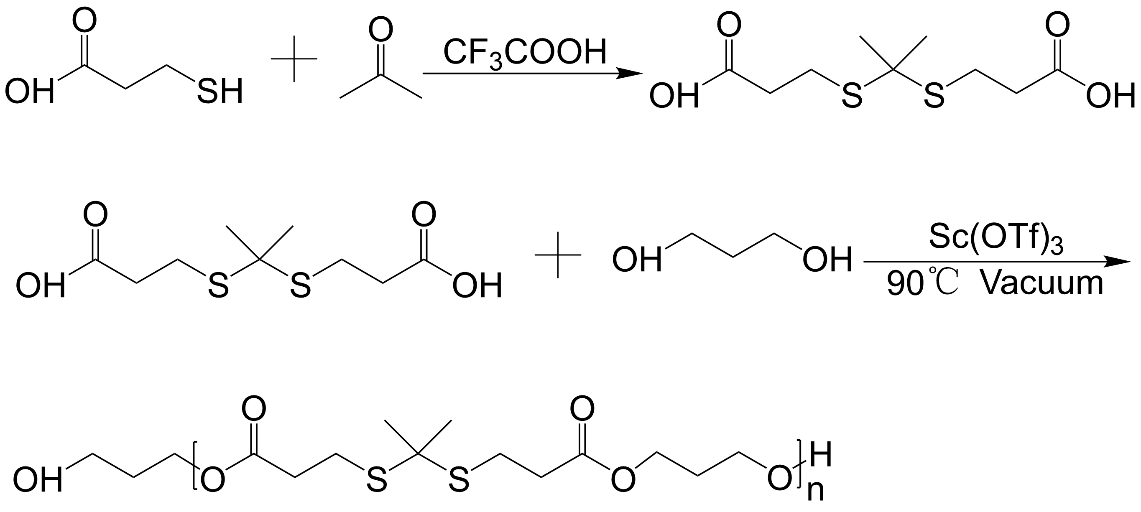


**Figure S1**. Synthesis route of PTK


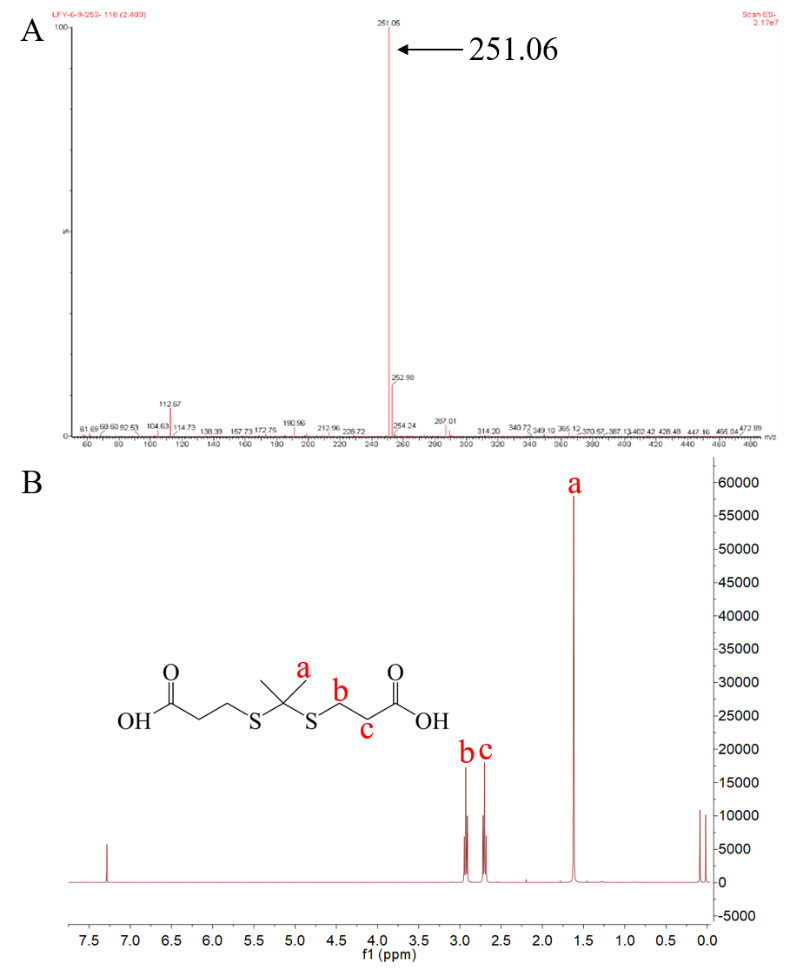


**Figure S2**. Mass Spectrum (A) and ^1^H NMR (B) of TK


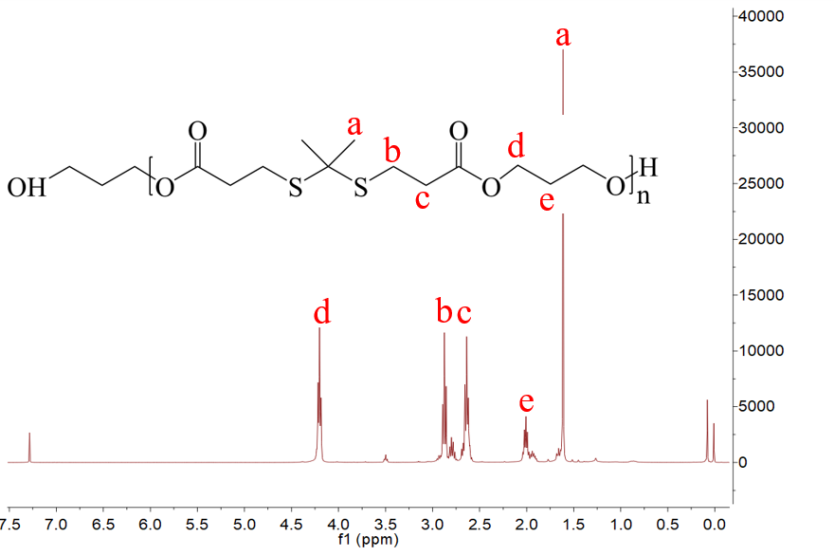


**Figure S3**. ^1^H NMR of PTK


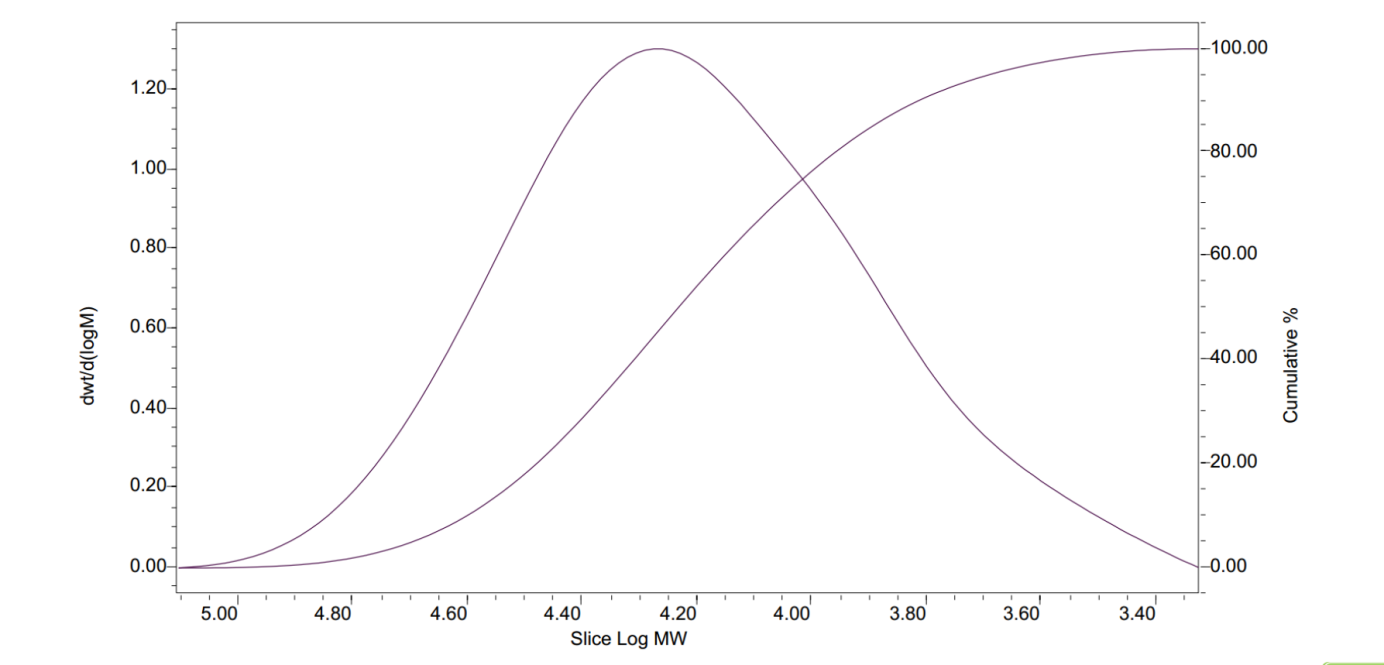


**Figure S4**. Gel permeation chromatography of PTK





**Figure S5**. Stability of CsA@PTK in PBS solution. n=3, mean±SD.

**
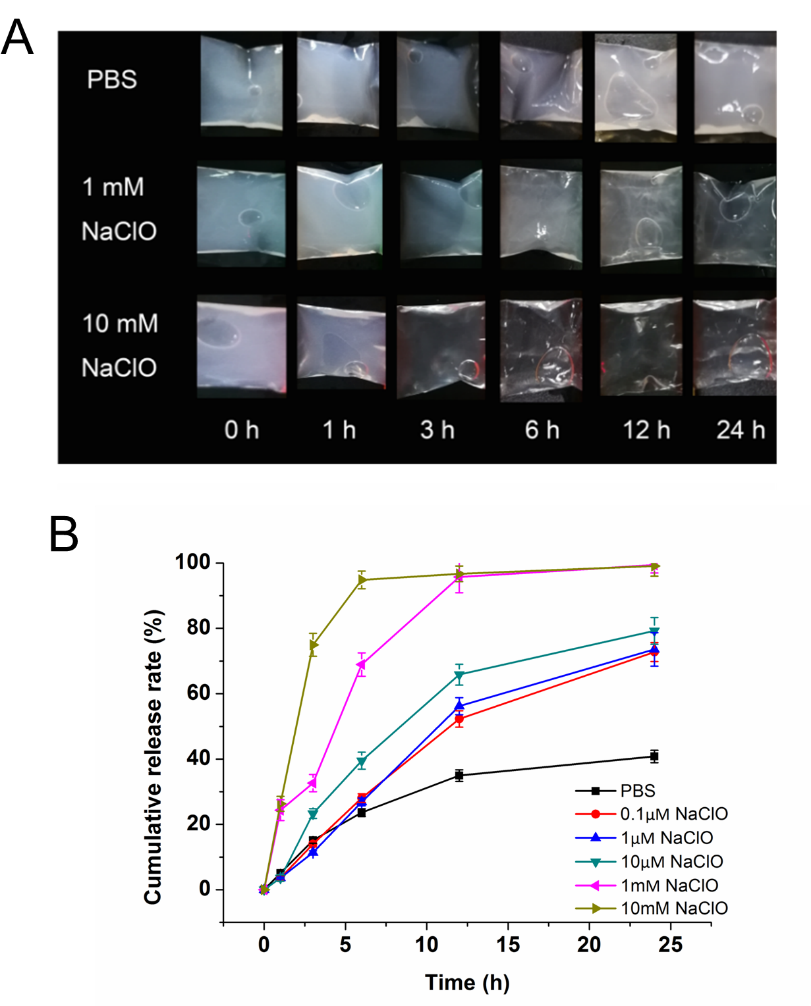
**

**Figure S6**. *In vitro* ROS responsibility of CsA@PTK. (A) The appearance changes of CsA@PTK in PBS (pH7.4) and NaClO-containing PBS (pH7.4) solution. (B) Cumulative drug release of CsA@PTK in PBS (pH7.4) and NaClO-containing PBS (pH7.4) solution. n=3, mean±SD.


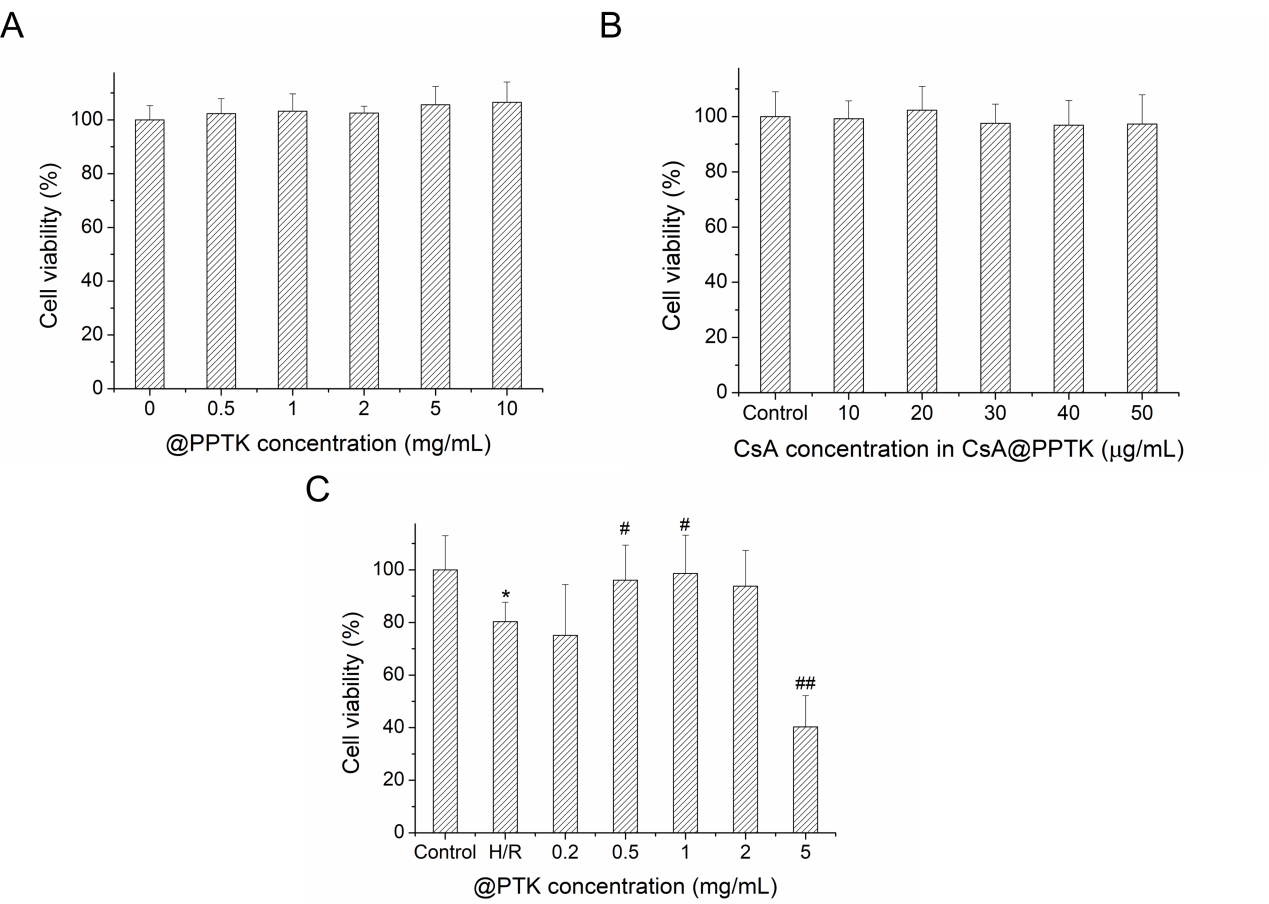


**Figure S7**. The cytotoxicity of CsA@PPTK. (A) The cytotoxicity of @PPTK on normal neonatal rat cardiomyocytes. (B) The cytotoxicity of CsA@PPTK on normal neonatal rat cardiomyocytes. (C) The cytotoxicity of @PTK on H/R injured H9c2 cells. n=6, mean±SD; ^*^P<0.05, ^**^P<0.01, ^***^P<0.001, versus control; ^#^P<0.05, ^##^P<0.01, versus H/R.

**
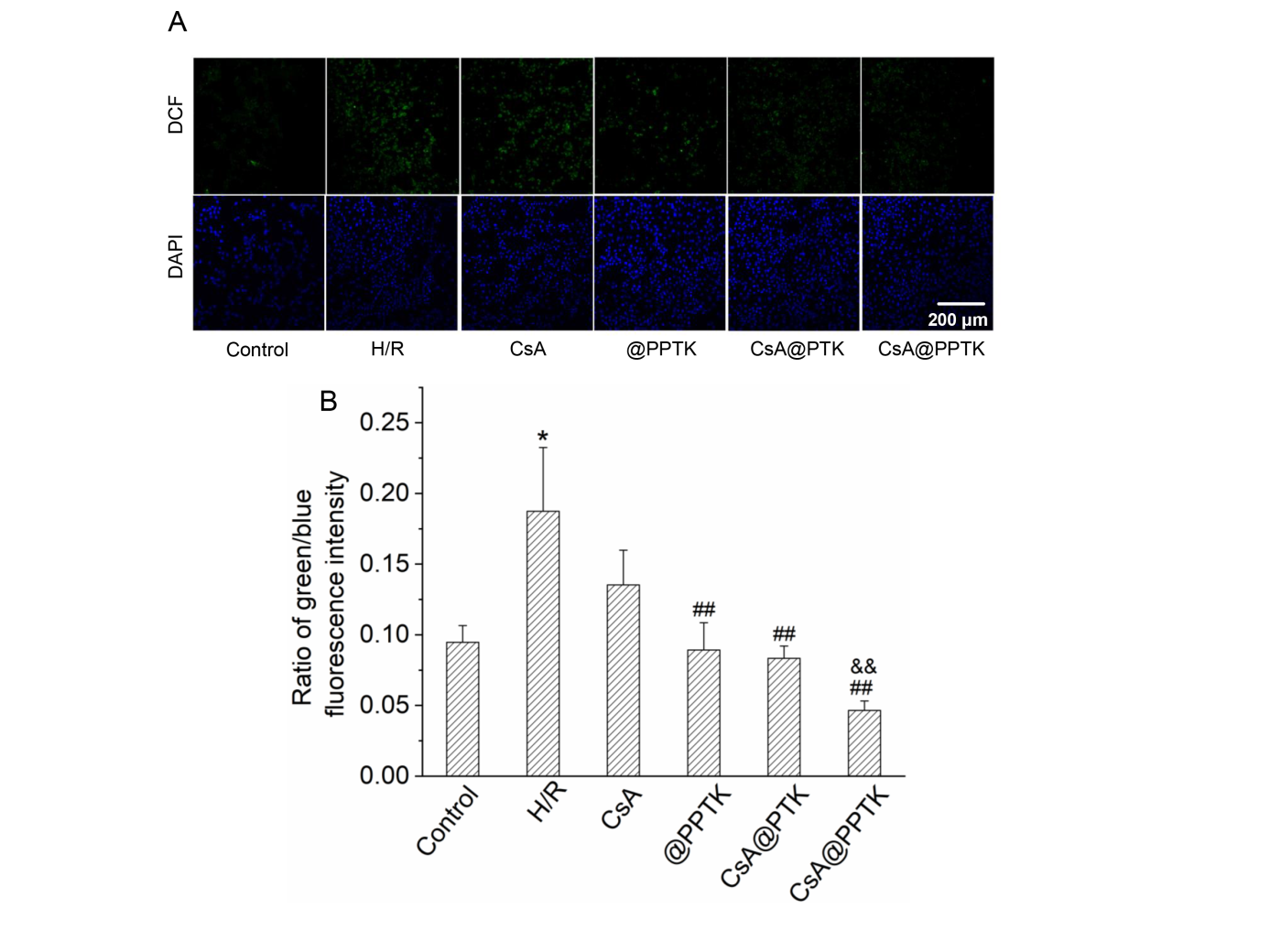
**

**Figure S8**. ROS scavenging effect of CsA@PPTK on H/R injured H9c2 cells. (A) The ROS in H/R injured H9c2 cells observed by fluorescence microscope. (B) Statistical results of ROS level in H/R injured H9c2 cell. n=3, mean±SD; ^*^p<0.05, versus control; ^##^p<0.01, versus H/R; ^&&^p<0.01, versus CsA@PTK.





**Figure S9**. Cellular uptake of CsA@PPTK by normal and H/R injured H9c2 cells. n=3, mean±SD; ^**^p<0.01, versus CsA@PPTK in H/R injured H9c2 cells at 0.5 h; ^#^p<0.05, versus CsA@PPTK in normal H9c2 cells at 2 h; ^&^p<0.05, versus CsA@PTK in H/R injured H9c2 cells at 2 h.





**Figure S10**. Effects of different uptake pathway inhibitors on the uptake of CsA@PPTK in H/R injured H9c2 cells. n=3, mean±SD; ^***^p<0.001, versus control (without the inhibitor).


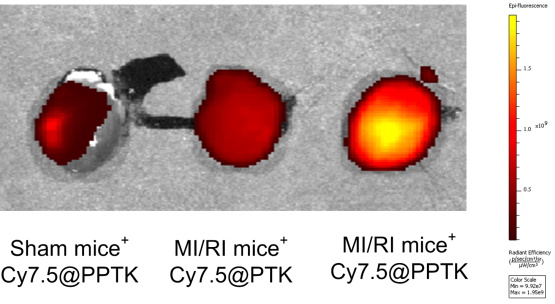


**Figure S11**. Distribution of Cy7.5@PPTK in heart of sham and MI/RI mice at 5 min after administration.


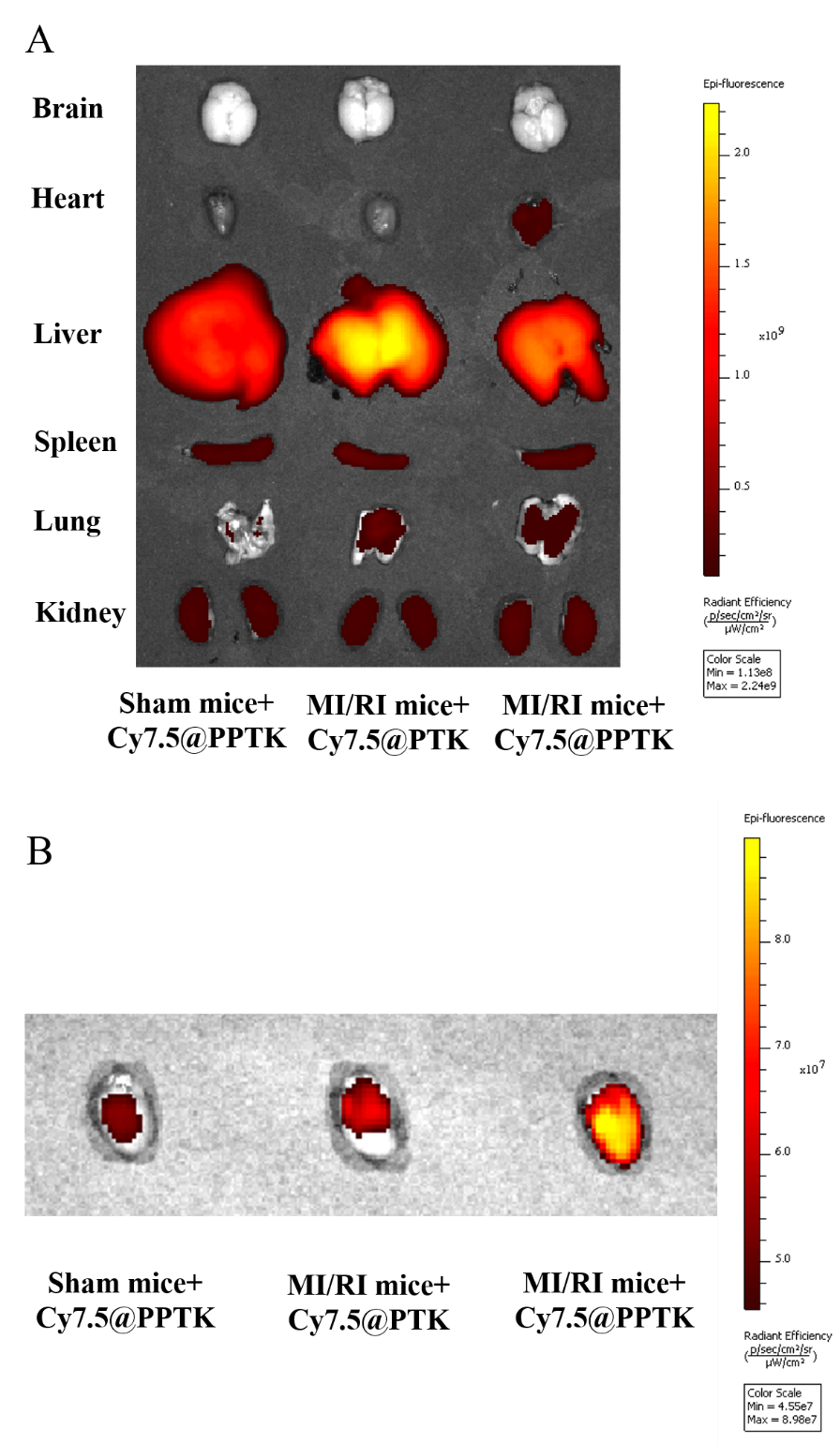


**Figure S12**. Distribution of Cy7.5@PPTK in main organs of sham and MI/RI mice at 24 h after administration.

**
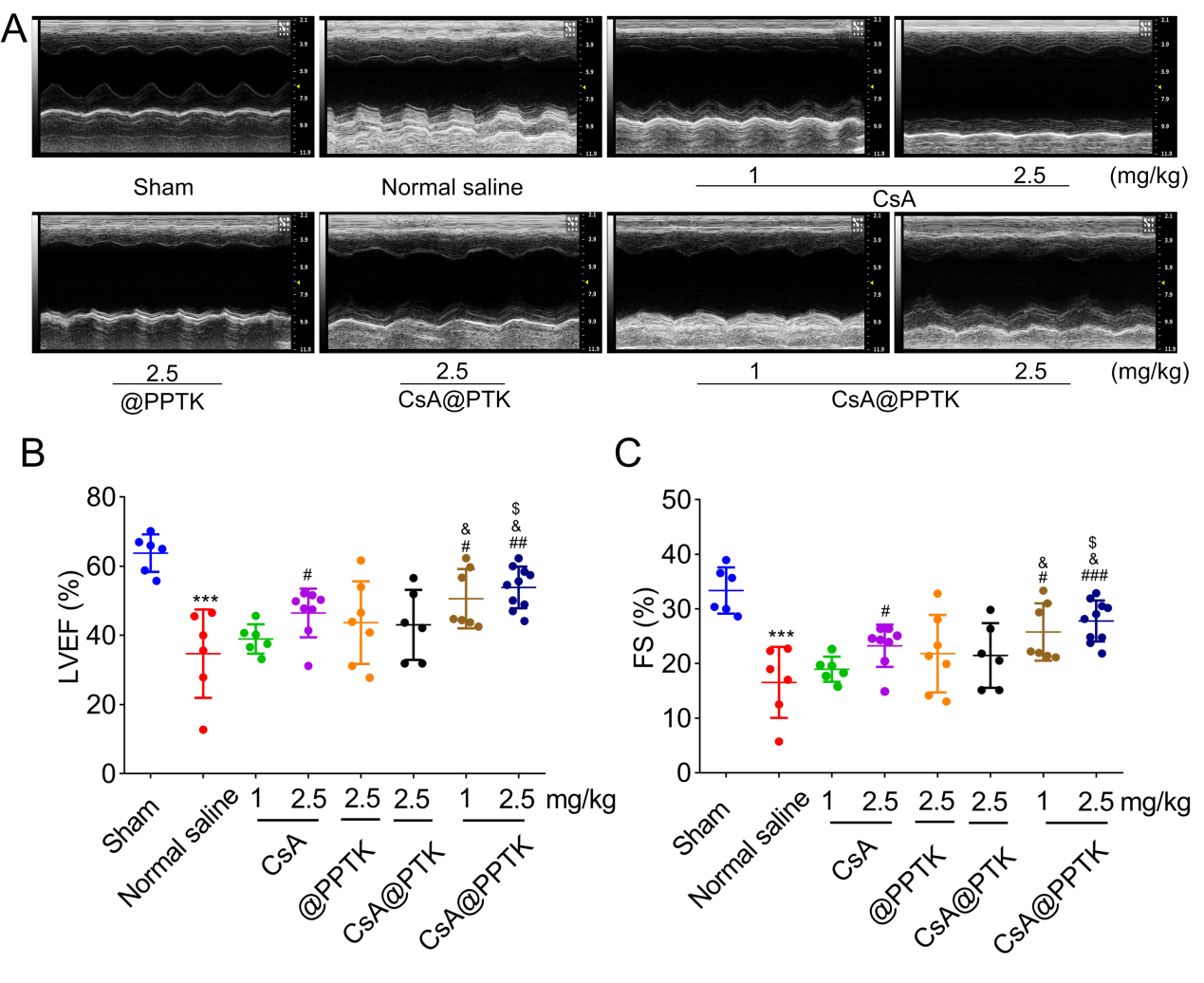
**

**FigureS13**. Left ventricular function of MI/RI mice at 70 days after reperfusion. (A) Representative echocardiography of MI/RI mice. (B) Statistical results of LVEF in different groups. (C) Statistical results of FS in different groups. n>5, mean±SD; ^***^p<0.001 versus sham; *^#^*p<0.05, *^##^*p<0.01, *^###^*p<0.001 versus normal saline; *^&^*p<0.05, versus CsA; *^$^*p<0.05, versus CsA@PTK.

**
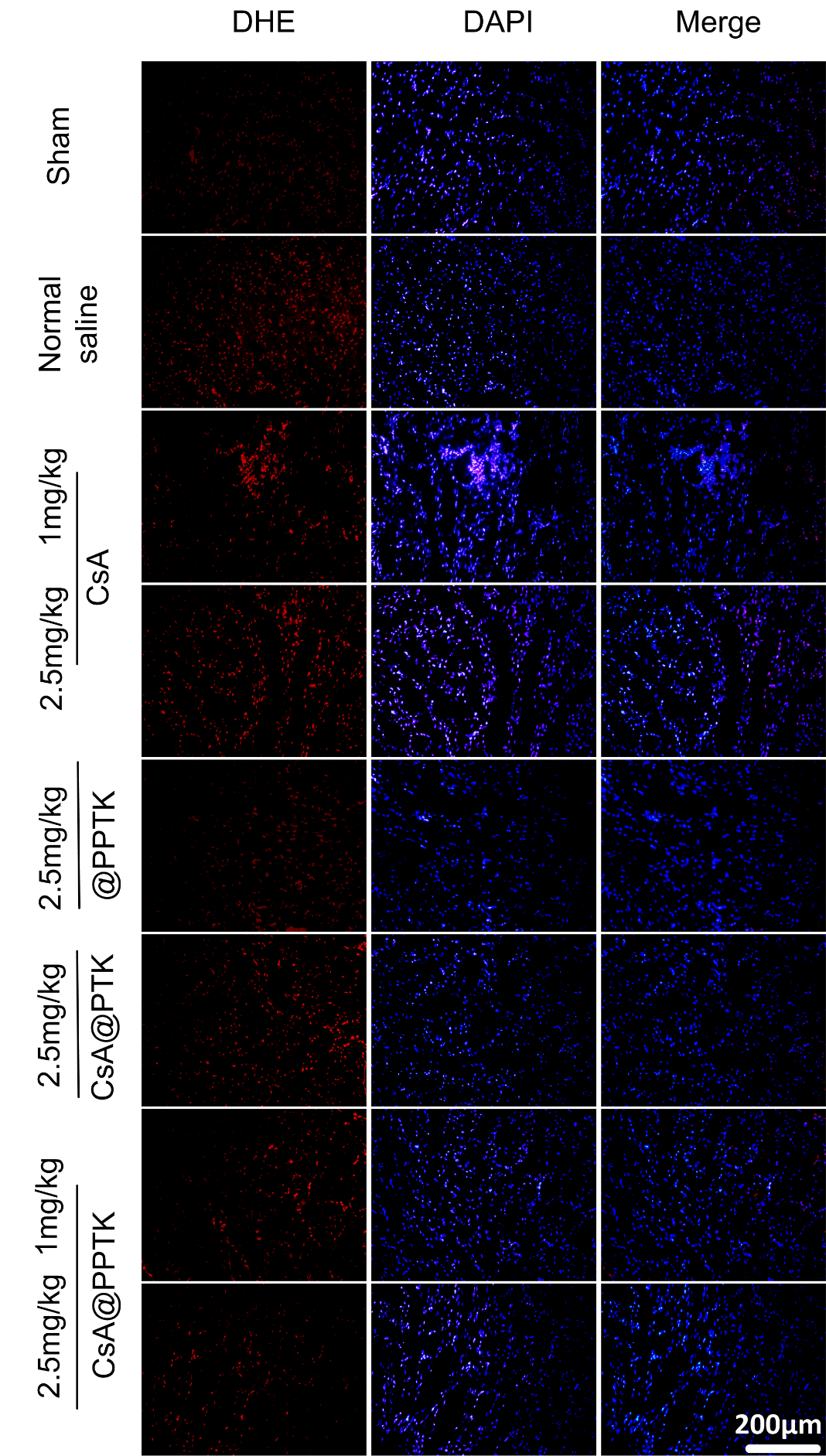
**

**Figure S14**. Representative image of ROS staining in heart tissue slice of MI/RI mice at 1 day after different treatment.


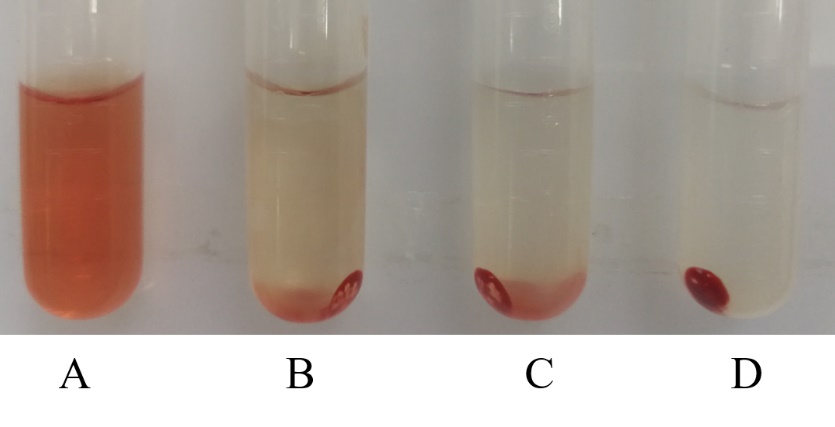


**Figure S15.** Hemolysis effect of deionized water (A), normal saline (B), CsA@PTK (C, 1 mg/mL) and CsA@PPTK (D, 1 mg/mL).


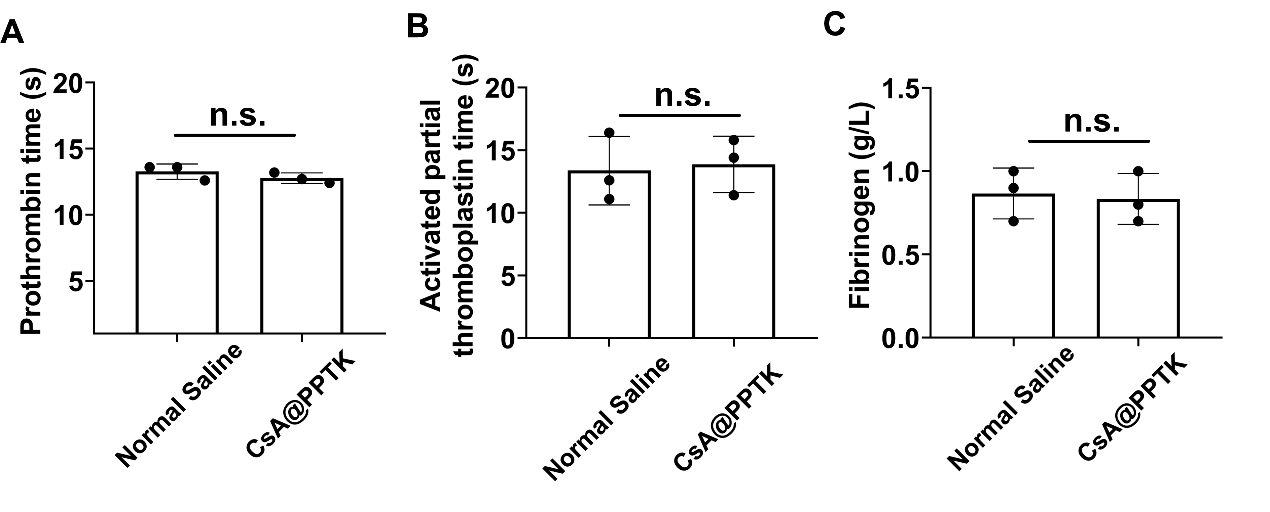


**Figure S16.** Evaluation of serum prothrombin time (PT) , activated partial thrombin time (APTT) and fibrinogen level (Fbg) at 28 days after treatment with normal saline and CsA@PPTK, n=3, mean±SD.


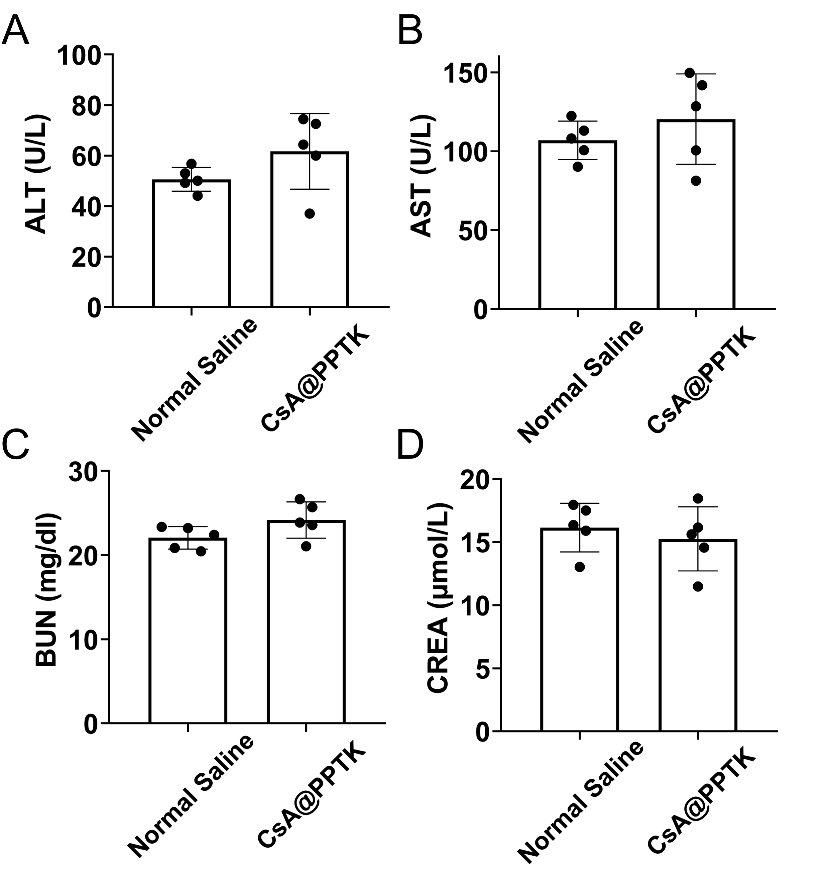


**Figure S17.** Evaluation of alanine aminotransferase (ALT), serum aspartate aminotransferase (AST), blood urea nitrogen (BUN) and creatinine (CREA) at 28 days after treatment with normal saline and CsA@PPTK, n=5, mean±SD.


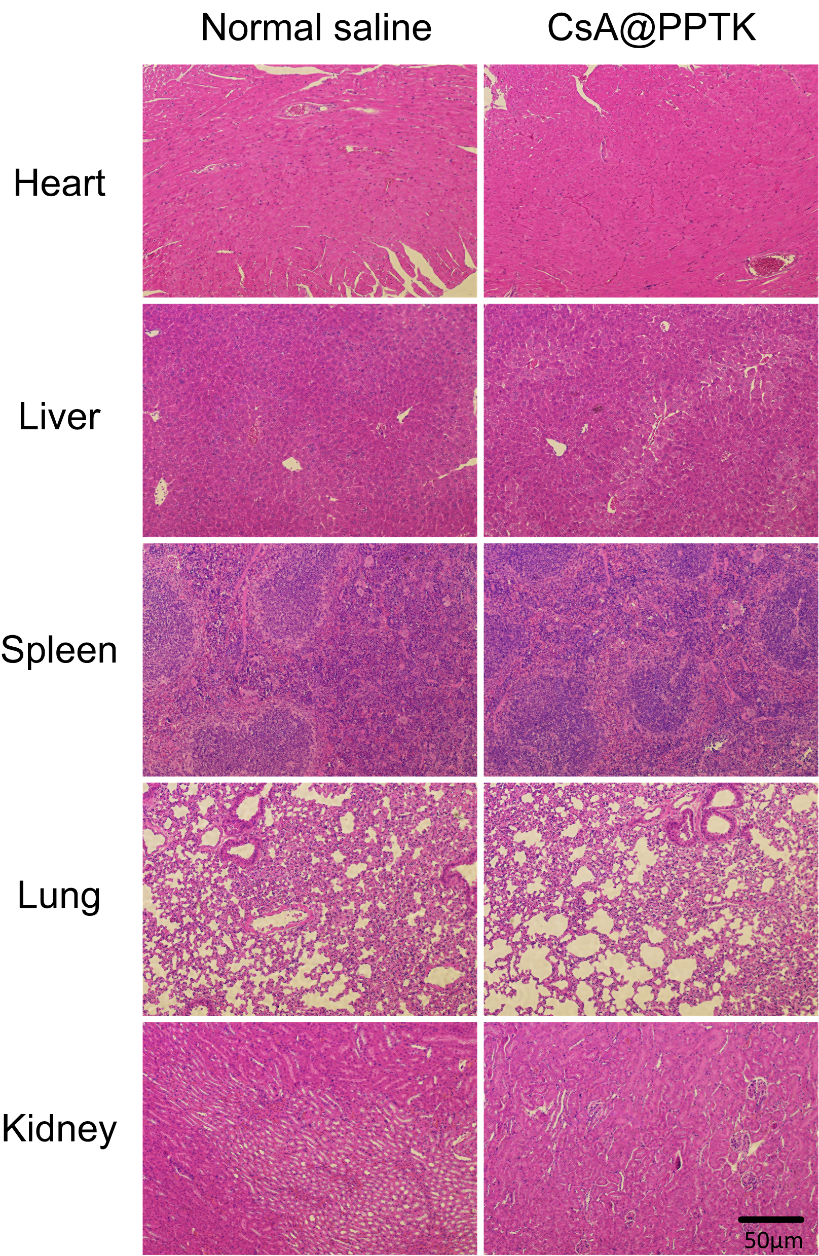


**Figure S18.** *H&E* staining of histological sections from major organs (heart, liver, spleen, lung, kidney) at 28 days after intravenous injection with normal saline or CsA@PPTK.


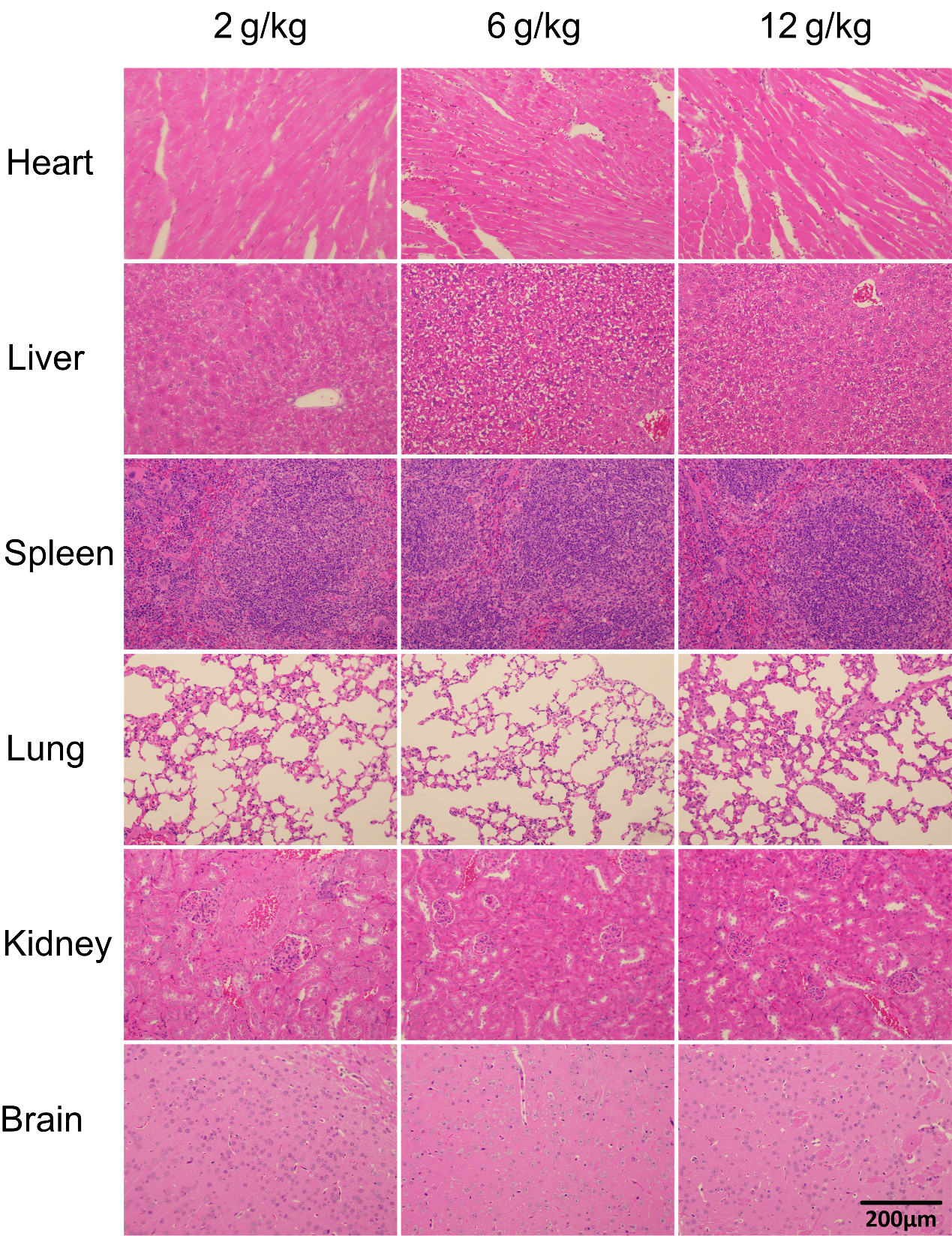


**Figure S19.** *H&E* staining of histological sections from major organs (heart, liver, spleen, lung, kidney and brain) at 14 days after intravenous injection of different dose of @PTK.


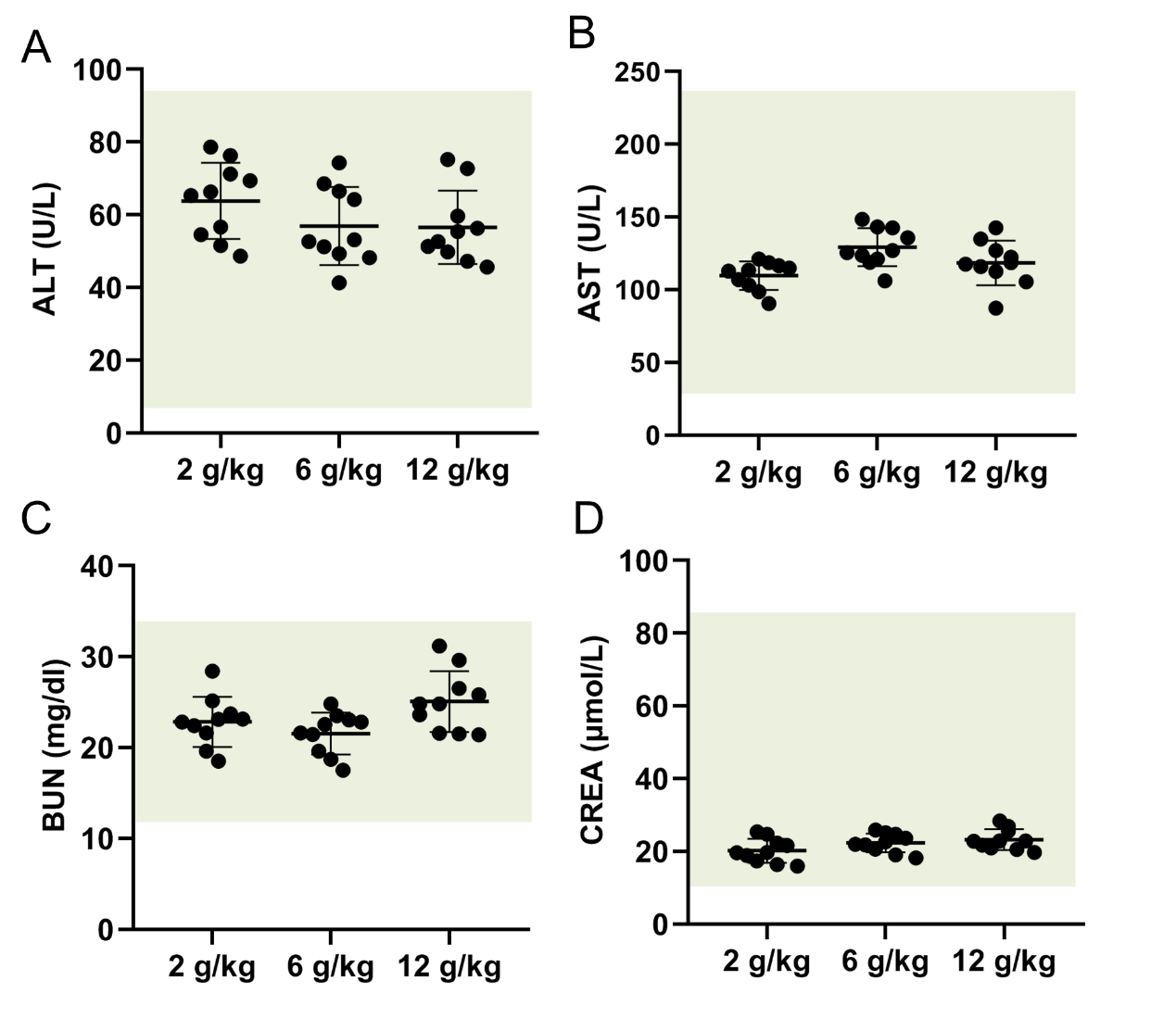


**Figure S20.** Evaluation of serum alanine aminotransferase (ALT), aspartate aminotransferase (AST), blood urea nitrogen (BUN) and creatinine (CREA)at 14 days after treatment with different dose of @PTK, n=10, mean±SD. Shadows represent the normal range of serum alanine aminotransferase (ALT), aspartate aminotransferase (AST), blood urea nitrogen (BUN) and creatinine (CREA) in mice.
